# Astrocytes in the Ventral Hippocampus Bidirectionally Regulate Innate and Stress‐Induced Anxiety‐Like Behaviors in Male Mice

**DOI:** 10.1002/advs.202400354

**Published:** 2024-08-09

**Authors:** Jing‐Ting Li, Shi‐Yang Jin, Jian Hu, Ru‐Xia Xu, Jun‐Nan Xu, Zi‐Ming Li, Meng‐Ling Wang, Yi‐Wen Fu, Shi‐Han Liao, Xiao‐Wen Li, Yi‐Hua Chen, Tian‐Ming Gao, Jian‐Ming Yang

**Affiliations:** ^1^ State Key Laboratory of Organ Failure Research Key Laboratory of Mental Health of the Ministry of Education Guangdong‐Hong Kong‐Macao Greater Bay Area Center for Brain Science and Brain‐Inspired Intelligence Guangdong Province Key Laboratory of Psychiatric Disorders Department of Neurobiology School of Basic Medical Sciences Southern Medical University Guangzhou Guangdong 510515 China

**Keywords:** anxiety, astrocytes, chemogenetics, fiber photometry, memantine, stress, ventral hippocampus

## Abstract

The mechanisms of anxiety disorders, the most common mental illness, remain incompletely characterized. The ventral hippocampus (vHPC) is critical for the expression of anxiety. However, current studies primarily focus on vHPC neurons, leaving the role for vHPC astrocytes in anxiety largely unexplored. Here, genetically encoded Ca^2+^ indicator GCaMP6m and in vivo fiber photometry calcium imaging are used to label vHPC astrocytes and monitor their activity, respectively, genetic and chemogenetic approaches to inhibit and activate vHPC astrocytes, respectively, patch‐clamp recordings to measure glutamate currents, and behavioral assays to assess anxiety‐like behaviors. It is found that vHPC astrocytic activity is increased in anxiogenic environments and by 3‐d subacute restraint stress (SRS), a well‐validated mouse model of anxiety disorders. Genetic inhibition of vHPC astrocytes exerts anxiolytic effects on both innate and SRS‐induced anxiety‐related behaviors, whereas hM3Dq‐mediated chemogenetic or SRS‐induced activation of vHPC astrocytes enhances anxiety‐like behaviors, which are reversed by intra‐vHPC application of the ionotropic glutamate N‐methyl‐d‐aspartate receptor antagonists. Furthermore, intra‐vHPC or systemic application of the N‐methyl‐d‐aspartate receptor antagonist memantine, a U.S. FDA‐approved drug for Alzheimer's disease, fully rescues SRS‐induced anxiety‐like behaviors. The findings highlight vHPC astrocytes as critical regulators of stress and anxiety and as potential therapeutic targets for anxiety and anxiety‐related disorders.

## Graphical abstract

Astrocytes in the ventral hippocampus (vHPC) display increased intracellular calcium levels in response to stress stimuli, leading to an ambient glutamate excess and enhanced anxiety behaviors. Disruption or enhancement of vHPC astrocytic calcium signals decreases or increases anxiety, respectively. In an anxiety mouse model, intra‐vHPC or systemic application of antagonists for the ionotropic glutamate N‐methyl‐d‐aspartate receptors rescues anxiety behaviors.

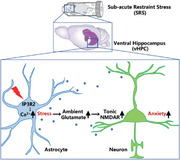

## 1 Introduction

Anxiety disorders are the most prevalent mental disorders with a lifetime prevalence of up to 33.7%.^[^ ^1^ ^]^ However, the pathological mechanisms underlying anxiety disorders are still largely unknown, and most pharmacological treatments pose clinical challenges due to the relatively modest efficacy, serious side effects, and a high relapse rate after medication discontinuation.^[^ ^2^ ^]^ Therefore, more efforts are needed to elucidate the pathogenesis of anxiety disorders and identify novel targets for anti‐anxiety treatments.

Stress is a key risk factor in the etiology of many neuropsychiatric disorders, in particular, mood and anxiety disorders (MAD). In addition to its central role in learning and memory, the hippocampus is one of the key brain regions involved in stress response and affect regulation in health and disease.^[^ ^3^ ^]^ In particular, the ventral subregion of the hippocampus (vHPC) is enriched in anxiety cells and essential for anxiety regulation through interacting with multiple cortical and subcortical regions such as the medial prefrontal cortex (mPFC), the lateral hypothalamic area, the lateral septum, and the bed nucleus of the stria terminalis.^[^ ^4^ ^]^ However, current research mainly focuses on the roles of hippocampal neurons in anxiety, with that of vHPC astrocytes largely unexplored.^[^ ^4^, ^5^ ^]^

Astrocytes are the most abundant cells in the brain, participate in most, if not all, of brain functions, and are critically involved in the pathological processes of various neuropsychiatric disorders.^[^ ^6^ ^]^ Through tightly enwrapping the vasculature with their endfeet and closely communicating with neurons and other non‐neuronal cells with fine processes in the brain, astrocytes are often regarded as a major hub of stress response and represent a nodal point that transduces stress signals such as the glucocorticoid hormones from the periphery to the brain.^[^ ^7^ ^]^ Mounting evidence from both animal and human studies has consistently demonstrated alterations of astrocyte number, density, morphology, or function in the brain, and astrocyte dysfunction is believed to contribute to the pathophysiology and possibly the pathogenesis of MAD.^[^ ^6^, ^8^ ^]^ For example, previous studies have demonstrated significant reductions in the number, density, and somatic volume of astrocytes in the PFC of MAD post‐mortem patients and disease models,^[^ ^9^ ^]^ and selective deletion of PFC astrocytes is sufficient to induce anxiety‐ and depressive‐like behaviors in rodents.^[^ ^10^ ^]^ However, the underlying mechanisms remain to be determined.

In this study, we explored the role and mechanism of vHPC astrocytes in the regulation of normal and pathological anxiety‐like behaviors and found that vHPC astrocytic activity was increased by anxiogenic stimuli, and inhibition and activation of vHPC astrocytes exerted anxiolytic and anxiogenic effects respectively on both innate and stress‐induced anxiety‐related behaviors. We conclude that vHPC astrocytes can bidirectionally regulate normal and pathological anxiety‐like behaviors.

## 2 Results

### 2.1 vHPC Astrocytic Activity was Increased in an Anxiogenic Environment

**Figure 1:**
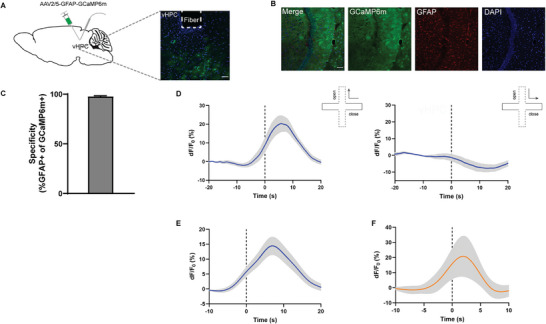
The activity of both ventral and dorsal hippocampal (vHPC and dHPC) astrocytes was increased in an anxiogenic environment. A) Schematic image of the AAV2/5‐GFAP‐GCaMP6m injection and fiber implantation in the vHPC. B) Fluorescence images illustrating virus expression in the vHPC. C) Percentage of GCaMP6m‐expressing astrocytes co‐labeled with GFAP. D) Representative vHPC Ca^2+^ traces during exploration of the open (left) or closed (right) arms in the elevated plus maze test (EPM). E) Same as (D) but during center exploration in the open field test (OFT). F) Same as (D) but for dHPC astrocytes. The dotted lines represent the time mice entered the open arms (D, left; F) or closed arms (D, right) in the EPM or the center in the OFT (E). n = 11 mice per group. Thick blue/orange lines indicate mean and shaded areas indicate SEM. Scale bars, 100 µm (A and B).

Astrocytes are electrically non‐excitable cells, and astrocytic activation is mainly manifested as an increase in intracellular Ca^2+^ levels, which are a primary signal through which astrocytes interact with neurons and other non‐neuronal cells in the brain.^[^ ^11^ ^]^ To determine whether the activity of vHPC astrocytes is associated with anxiogenic environments, we measured the levels of Ca^2+^ activity in vHPC astrocytes in mice freely exploring in the elevated plus maze (EPM) and the open field test (OFT), two widely used behavioral tests to assess anxiety‐like behaviors in rodents, by using in vivo fiber photometry calcium imaging. For this, adeno‐associated virus (AAV)2/5 encoding the fluorescent biomarker GCaMP6m under the control of an astrocyte‐specific GFAP promotor (AAV2/5‐GFAP‐GCaMP6m) was injected into the vHPC and an optical fiber was implanted at the same site (**Figure** **1**A). Three weeks after virus injection, immunostaining revealed that GCaMP6m expression was detected almost exclusively in astrocytes as 97% of GCaMP6m‐positive cells were co‐labeled with the astrocyte marker GFAP (Figure 1B,C), indicating a high specificity of GCaMP6m expression in astrocytes.

In the EPM, we found that vHPC astrocytes exhibited a significant increase in Ca^2+^ activity when mice approached and explored the anxiogenic open‐arm compartment but not the anxiolytic closed‐arm compartment (Figure 1D). Similar results were also observed when mice explored the center zone in the OFT (Figure 1E). The change of astrocytic Ca^2+^ activity in the EPM was not vHPC‐specific as astrocytes in the dorsal HPC (dHPC) also displayed similar changes (Figure 1F). These results obtained from population recordings of a small cluster of GCaMP6m‐expressing astrocytes in freely moving mice are similar to those obtained from individual dHPC astrocytes through two‐photon Ca^2+^ imaging in head‐fixed mice exposed to a virtual reality environment emulating the EPM.^[^ ^5^ ^]^ Together, these findings suggest that exposure to anxiogenic environments increased Ca^2+^ activity of hippocampal astrocytes that may reflect an anxiety state of mice. Based on abundant previous findings highlighting a critical role for the vHPC in the actions of anxiety and a lack of knowledge of vHPC astrocytic contribution to these processes,^[^ ^4^, ^5^ ^]^ in subsequent experiments, we focused on assessing the regulation of innate and stressed‐induced anxiety‐like phenotypes by vHPC astrocytes.

### 2.2 Conditional Astrocytic *Itpr2* Knockout in the vHPC Produced Anxiolytic Effects

**Figure 2:**
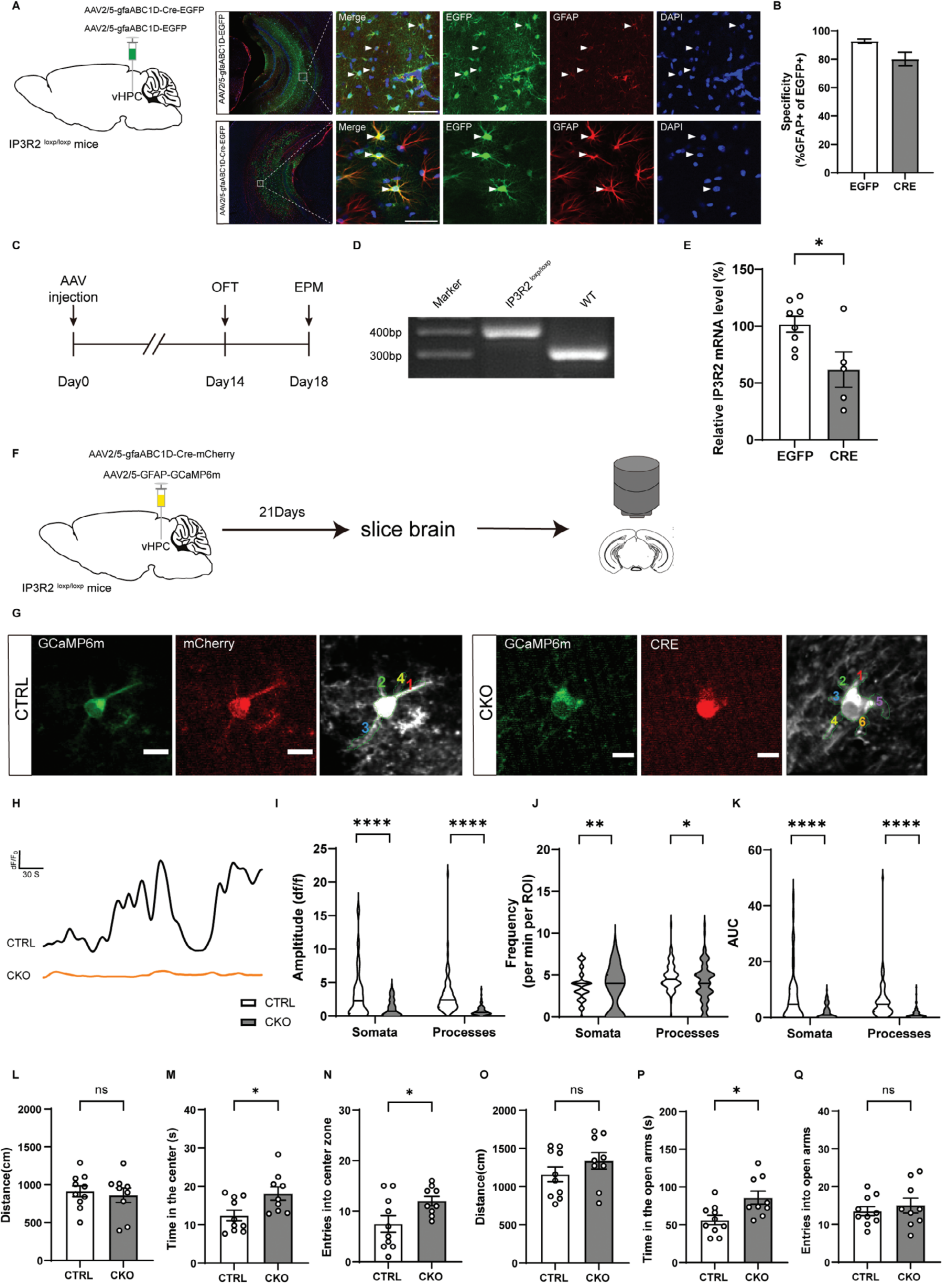
Conditional astrocytic *Itpr2* knockout in the vHPC produced anxiolytic effects. A) Representative images showing AAV2/5‐gfaABC1D‐Cre‐EGFP (CKO) and AAV2/5‐gfaABC1D‐EGFP (CTRL) virus expression three weeks after injection into the vHPC of IP3R2^loxp/loxp^ mice. B) Percentage of EGFP‐expressing astrocytes co‐labeled with GFAP. C) Experimental timeline for behavioral tests after AAV injection. D) Genotyping of IP3R2^loxp/loxp^ (middle) and wildtype (right) mice by PCR analysis of genomic DNA. E) Reduced IP3R2 mRNA level in CKO mice compared to CTRL mice. F) Schematic experimental approach. G) Two‐photon calcium imaging of an mCherry&GCaMP6m‐expressing astrocyte (left) and a Cre&GCaMP6m‐expressing astrocyte (right). H) Representative fluorescence traces from IP3R2 CTRL and CKO mice. I–K) Summary of the results from all imaged cells. *n* = 52/41 cells from three mice for somata analysis, *n* = 84/98 cells from three mice for processes analysis. L–N) CKO mice (*n* = 9) showed increased center zone exploration compared with CTRL mice (*n* = 10) during the OFT. O–Q) CKO mice (*n* = 9) showed increased open‐arm exploration compared with CTRL mice (*n* = 10) during the EPM. Scale bars, 100 µm (A) and 10 µm (G). Data are presented as mean ± SEM; two‐tailed unpaired *t*‐test. *P < 0.05, **P < 0.01, ***P < 0.001, ****P < 0.0001, ns, no significance. Each data point represents an individual mouse.

Intracellular Ca^2+^ increase in astrocytes in the brain is mainly mediated by type 2 inositol 1,4,5‐trisphosphate receptors (IP3R2).^[^ ^6c^ ^]^ To determine whether IP3R2 knockout in astrocytes in adult mice has any effect on innate anxiety‐like behaviors, we generated astrocyte‐specific IP3R2 conditional knockout mice (CKO mice) by expressing Cre recombinase under the control of an astrocyte‐specific gfaABC1D promoter in the vHPC of IP3R2^loxp/loxp^ mice (**Figure** **2**A,B), and then anxiety‐like behaviors were tested (Figure 2C). To ascertain successful *Itpr2* knockout in vHPC astrocytes, the following two experiments were done. First, IP3R2^loxp/loxp^ mice were confirmed by PCR of genomic DNA (Figure 2D), and a significant reduction of IP3R2 mRNA expression in the vHPC was confirmed by the qRT‐PCR in CKO mice compared with controls (Figure 2E). Second, we performed two‐photon calcium imaging by co‐expressing Cre recombinase and GCaMP6m in vHPC astrocytes in IP3R2^loxp/loxp^ mice, and the basic properties of the Ca^2+^ signals in hippocampal slices were analyzed and quantified (Figure 2F,G). We found that the amplitude, frequency and AUC (area under curve) of Ca^2+^ signals in astrocytic somata and processes were markedly decreased in CKO mice compared to control mice (Figure 2H–K). Together, these data demonstrate that astrocytic *Itpr2* knockout in the vHPC dramatically decreased astrocytic calcium signals. In the OFT, compared to the control group, CKO mice exhibited a significant increase in center zone exploration and the number of center entries (Figure 2L–N). During the EPM, the time CKO mice spent in the open arms was significantly increased compared to control mice (Figure 2O–Q). Of note, in vivo calcium imaging showed that vHPC astrocytic Ca^2+^ activity remained unchanged during CKO mice entry into either the open or closed arms in the EPM (Figure S1A,B, Supporting Information). These findings indicate that vHPC astrocytic *Itpr2* knockout enhanced anxiolytic behaviors, possibly by disrupting Ca^2+^ signals in astrocytes.

To exclude the possibility that the above finding was confounded by potential leaky expression of Cre recombinase in neurons, we then stereotaxically injected AAV2/9‐CamkII‐Cre‐EGFP or AAV2/9‐CamkII‐EGFP into the vHPC of IP3R2^loxp/loxp^ mice to generate neuron‐specific IP3R2 conditional knockout mice (nCKO mice) (Figure S2A, Supporting Information). Immunohistochemical staining showed EGFP‐expressing cells were co‐labeled with the neuronal marker NeuN (Figure S2B, Supporting Information). After verifying the knockout efficiency of IP3R2 mRNA expression in the vHPC by the qRT‐PCR (Figure S2C, Supporting Information), the OFT and EPM were performed. We found no difference in behavioral measures of anxiety between nCKO mice and controls (Figure S2D–I, Supporting Information). Notably, neuron‐specific IP3R2 conditional knockout did not affect the increase in vHPC astrocytic Ca^2+^ activity during mice entry into the open arm in the EPM (Figure S1C, Supporting Information). These data suggest that conditional astrocytic but not neuronal *Itpr2* knockout in the vHPC produced anxiolytic effects.

These findings demonstrate that genetic inhibition of vHPC astrocytes exerted anxiolytic effects in stress‐naive mice, suggesting that vHPC astrocytes are critical for maintaining an appropriate baseline level of innate anxiety, with the underlying mechanisms awaiting further studies.

### 2.3 Chemogenetic Activation of vHPC Astrocytes Induced Anxiety‐Like Behaviors

**Figure 3:**
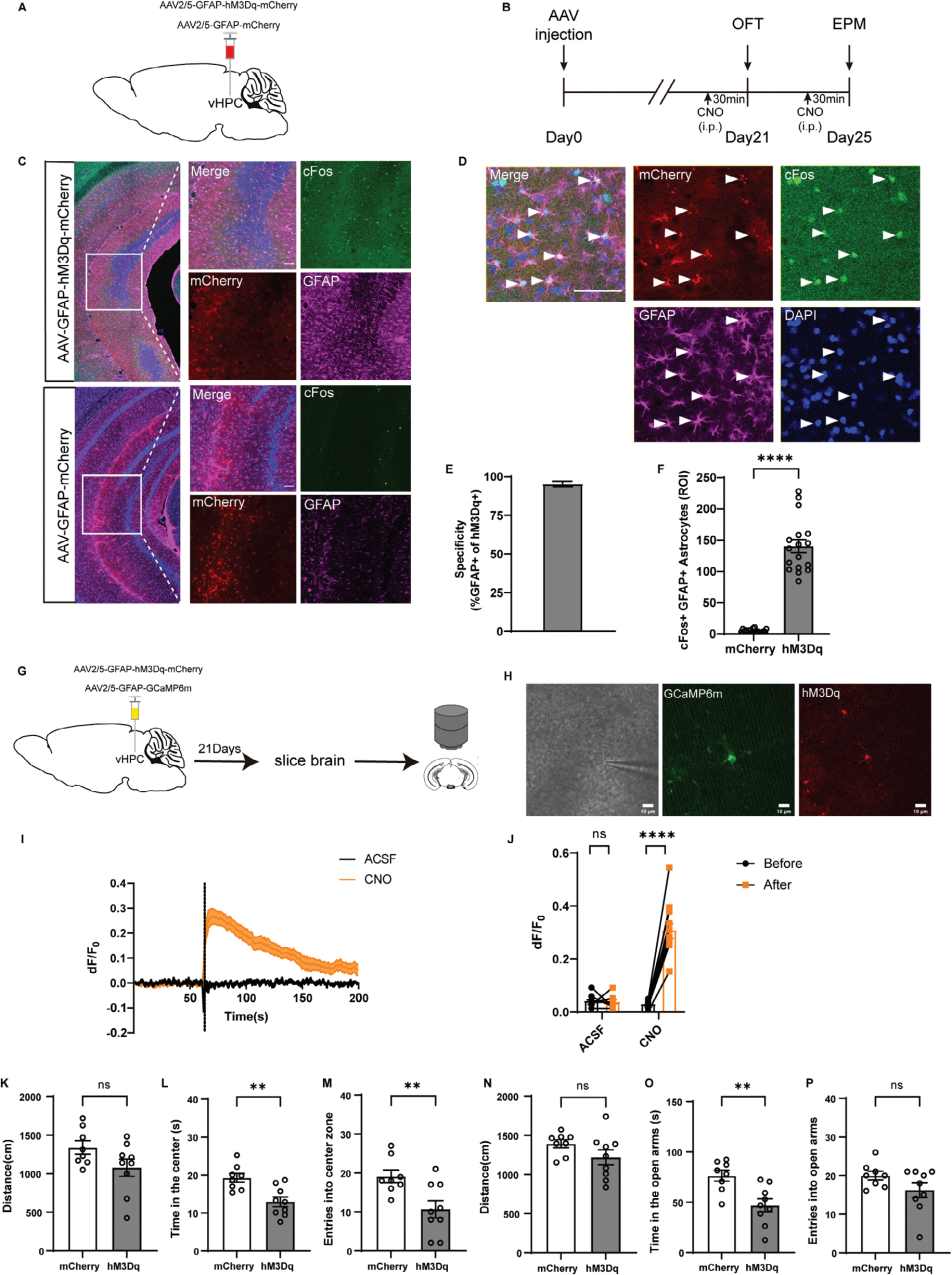
Acute chemogenetic activation of vHPC astrocytes enhanced anxiety‐like behaviors. A) Schematic showing injection of AAV2/5‐GFAP‐hM3Dq‐mCherry and AAV2/5‐GFAP‐mCherry into the vHPC. B) Experimental scheme. C) Fluorescence images illustrating virus expression in the vHPC. D) Representative images showing cFos expression in vHPC astrocytes expressing hM3Dq after i.p. injection of CNO. Arrow heads indicate astrocytes expressing hM3Dq co‐labeled with cFos and GFAP. E) Percentage of GFAP‐labeled astrocytes co‐labeled with hM3Dq. F) Number of cFos‐expressing GFAP‐labeled astrocytes in hM3Dq group (*n* = 17 slices from 5 mice) compared with mCherry group (*n* = 18 slices from 5 mice). G) Schematic experimental approach. H) Two‐photon calcium imaging of an hM3Dq‐ and GCaMP6m‐expressing astrocyte, with an adjacently placed glass pipette. I) Representative fluorescence traces from hM3Dq‐ and GCaMP6m‐expressing astrocytes treated with ACSF or 5 × 10^−3^ m CNO. The dotted line represents the time ACSF or 5 × 10^−3^ m CNO was applied. J) Summary of the results from all imaged cells (*n* = 8/12 cells from 3/4 mice). K–P) Chemogenetic activation of vHPC astrocytes decreased the center exploration in the OFT (K–M) and open‐arm exploration in the EPM (N–P), *n* = 8, 9. Data are presented as mean ± SEM; two‐tailed unpaired *t* test. ns, no significance. Each data point represents an individual mouse. Scale bars, 100 µm (C and D) and 10 µm (H). Data are presented as mean ± SEM; Mann–Whitney *U*‐test (F), two‐tailed paired *t*‐test (H), two‐tailed unpaired *t* test (K–P). *P < 0.05, **P < 0.01, ***P < 0.001, ****P < 0.0001, ns, no significance. Each data point represents an individual mouse.

Astrocytes communicate with neurons mainly by expressing various Gq‐coupled metabotropic receptors (GPCR) that respond to neurotransmitters or neuromodulators to induce an increase in intracellular Ca^2+^ levels.^[^ ^12^ ^]^ Based on the capacity of Gq‐coupled DREADD (hM3Dq) to induce Ca^2+^ elevation in astrocytes,^[^ ^13^ ^]^ we next expressed Gq‐coupled hM3Dq, which is exclusively activated by the designer drug clozapine‐N‐oxide (CNO), in vHPC astrocytes to mimic endogenous Gq‐GPCR activation and test whether chemogenetic activation of vHPC astrocytes was sufficient to cause anxiety‐like behaviors (**Figure** **3**B). AAV2/5‐GFAP‐hM3Dq‐mCherry or AAV2/5‐GFAP‐mCherry (control virus) was delivered into the vHPC stereotaxically (Figure 3A). Immunostaining results showed that hM3Dq expression was limited to GFAP‐positive astrocytes with a high specificity (>95% hM3Dq‐expressing cells were also GFAP‐positive) (Figure 3C–E). To confirm that hM3Dq did activate astrocytes upon CNO application, two experiments were done. First, we co‐stained for the immediate‐early gene cFos and astrocytic marker GFAP 30 min after CNO administration (3 mg kg^−1^, intraperitoneally, i.p.) and found co‐staining of cFos with hM3Dq‐mCherry‐ and GFAP‐expressing cells, but not with mCherry‐ and GFAP‐expressing cells (Figure 3C,D,F). Second, we performed two‐photon calcium imaging by co‐expressing hM3Dq and GCaMP6m in vHPC astrocytes and applying CNO (5 × 10^−3^ m) or ACSF locally to hM3Dq‐expressing cells with a glass pipette placed adjacently (Figure 3G,H) in hippocampal slices. As expected, CNO application induced increased Ca^2+^ signals while ACSF failed to trigger any Ca^2+^ signals (Figure 3I,J). Thus, these data demonstrate that hM3Dq can induce specific activation of vHPC astrocytes upon CNO application.

To test whether chemogenetic activation of vHPC astrocytes modulated anxiety‐related phenotypes, a single i.p. injection of CNO (3 mg kg^−1^) was applied 30 min before behavioral tests. Compared to the mCherry controls, mice in the hM3Dq group showed decreased center zone exploration and lower probability of center zone entry in the OFT (Figure 3K–M), and spent less time in EPM open arms (Figure 3N–P). These effects were not due to the application of the CNO per se, as the same dose of CNO had no effect on anxiety‐like behaviors in an additional cohort of mice without Gq‐DREADD expression (Figure S3, Supporting Information). These results indicate that chemogenetic activation of vHPC astrocytes enhanced anxiety‐like behaviors.

Previous studies have shown that Gq‐DREADD activation of cortical astrocytes elevates baseline Ca^2+^ level lasting for hours.^[^ ^14^ ^]^ To test whether chemogenetic activation of vHPC astrocytes produced long‐lasting effect on anxiety‐like behaviors, behavioral tests were done 24 h after CNO application in another cohort of mice (Figure S4A, B, Supporting Information), and no effect was found (Figure S4C–H).

A recent study showed that ChR2‐mediated optogenetic activation of either dorsal or ventral hippocampal astrocytes leads to a widespread cFos expression throughout the whole hippocampus.^[^ ^5^ ^]^ To assess the effect of chemogenetic activation of vHPC astrocytes on neuronal activity, cFos immunostaining was done in the dorsal and ventral hippocampus. We found that CNO delivery resulted in a significant increase in cFos expression in ventral (Figure S5A,B, Supporting Information) but not dorsal (Figure S5C,D, Supporting Information) hippocampal neurons. Taken together, our results show that acute chemogenetic activation of vHPC astrocytes produced an anxiogenic effect possibly through activation of local neurons in the ventral hippocampus.

### 2.4 Blockade of the N‐Methyl‐d‐Aspartate Receptor (NMDAR) Rescued Anxiety‐Like Behaviors in Mice with vHPC Astrocytes Chemogenetically Activated

**Figure 4:**
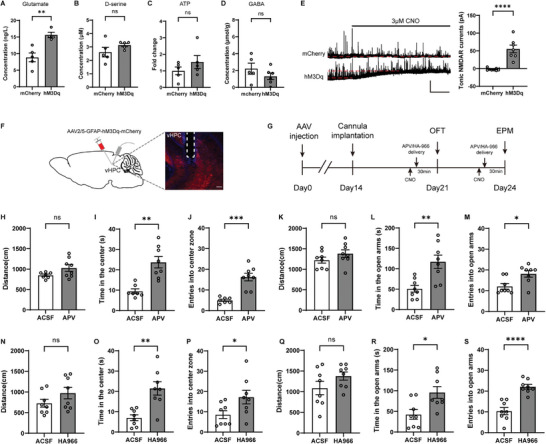
Anxiety‐like behaviors induced by chemogenetic activation of vHPC astrocytes were reversed by intra‐vHPC application of the N‐methyl‐d‐aspartate receptor (NMDAR) antagonists APV and HA‐966. A–D) The levels of glutamate (A, *n* = 5, 4), d‐serine (B, *n* = 5, 5), ATP (C, *n* = 5, 5), and GABA (D, *n* = 5, 6) after chemogenetic activation of vHPC astrocytes from mCherry group and hM3Dq group. E) Sample traces of tonic NMDAR current recordings from vHPC CA1 pyramidal neurons with astrocytes expressing hM3Dq (left) and quantification of tonic NMDAR currents (right, *n* = 8, 7 cells from 6 mice). Black lines indicate the changes of baseline induced by application of the CNO (3 × 10^−6^ m). F) Confocal image showing the placement of the cannula above the vHPC and the expression of AAV2/5‐GFAP‐hM3Dq‐mCherry in the vHPC. G) Experimental timeline. H‐M APV (5 × 10^−3^ m) rescued anxiety‐like behaviors in the OFT (H–J, *n* = 7, 8) and EPM (K–M, *n* = 8, 8) in astrocyte‐activated animals. N–S) (R)‐(+)‐HA‐966 (50 × 10^−3^ m) rescued anxiety‐like effects in the OFT (N–P) and EPM (R–T, *n* = 8, 8) in astrocyte‐activated mice. Scale bars: 2 min, 50 pA (E) and 100 µm (F). Data are presented as mean ± SEM; two‐tailed unpaired *t*‐test. *P < 0.05, **P < 0.01, ***P < 0.001, ****P < 0.0001, ns, no significance. Each data point represents an individual mouse (A–D, H–S).

Astrocytes can sense neuronal activity and release multiple gliotransmitters, which in turn, regulate neuronal activity and ultimately behaviors.^[^ ^15^ ^]^ We first measured the levels of glutamate, GABA, ATP, and d‐serine, four well‐recognized gliotransmitters,^[^ ^15^ ^]^ in vHPC slices collected from mice expressing hM3Dq and subjected to CNO injection. Surprisingly, we found that only the level of glutamate but not GABA, ATP, or d‐serine was increased (**Figure** **4**A–D). These results suggest that chemogenetically activated vHPC astrocytes may regulate anxiety‐like behaviors by increasing ambient glutamate levels.

To address whether ambient glutamate following astrocyte activation increased tonic NMDAR currents in adjacent neurons, we recorded vHPC CA1 pyramidal neurons at a holding potential of +40 mV with hM3Dq expressed in vHPC astrocytes (Figure 4E). Application of the CNO (3 × 10^−6^ m) induced an increase in baseline NMDAR currents (Figure 4E), indicating ambient glutamate was increased by chemogenetically activated vHPC astrocytes.

To test whether glutamate increase is responsible for the behavioral changes observed upon chemogenetic activation of vHPC astrocytes, we delivered APV (5 × 10^−3^ m), an NMDAR antagonist, into the vHPC 30 min prior to the OFT and EPM (Figure 4F,G). 5 × 10^−3^ m APV was used as it had no significant effect on the locomotor activity in the OFT (Figure S6A, Supporting Information) or anxiety‐related behaviors in wild‐type mice (Figure S6B–G, Supporting Information). hM3Dq was expressed in vHPC astrocytes as previously described and guide cannulas were implanted in the vHPC (Figure 4F). ACSF or APV was delivered to the vHPC immediately after i.p. application of CNO (Figure 4G). We found that APV application before behavioral tests significantly ameliorated the anxiety‐like behaviors induced by chemogenetic activation of vHPC astrocytes (Figure 4H–M). Memantine is an NMDAR antagonist approved by the U.S. FDA for the treatment of Alzheimer's disease. To further confirm the contribution of the NMDAR to the anxiogenic effects of vHPC astrocyte activation, we delivered memantine (50 × 10^−3^ m) locally to the vHPC before behavioral tests and found an improvement in anxiety‐like behaviors (Figure S7, Supporting Information). Taken together, these data indicate that delivery of the NMDAR antagonists APV or memantine into the vHPC rescued the anxiogenic effects of vHPC astrocytic activation.

Activation of NMDAR requires binding of their ligand glutamate and co‐agonist, glycine or d‐serine. To further ascertain the involvement of NMDAR in mediating anxiety‐like behaviors, we tested the effect of NMDAR glycine site blocker (R)‐(+)‐HA‐966 on the increased anxiety‐like behaviors caused by chemogenetic activation of vHPC astrocytes (Figure 4G). We chose a dose of HA966 (50 × 10^−3^ m) that had no effect on baseline anxiety level in wild‐type mice (Figure S8, Supporting Information). As expected, we found that application of HA966 (50 × 10^−3^ m) in the vHPC induced similar anxiolytic effects as APV treatment, as it rescued anxiety phenotypes caused by astrocytic activation (Figure 4N–S), confirming the involvement of the NMDAR in anxiety‐related behaviors induced by chemogenetic activation of vHPC astrocytes.

To determine if, besides NMDAR, other glutamate receptors also play a part in mediating the enhanced anxiety levels caused by vHPC astrocytic activation, we chose to target the metabotropic glutamate receptor 5 (mGluR5), which has been shown to mediate social isolation‐induced anxiety‐like behaviors in our previous study.^[^ ^16^ ^]^ We found that intra‐vHPC application of the mGluR5 antagonist MPEP (20 × 10^−6^ m) failed to improve anxiety‐like behaviors in vHPC astrocytes‐activated mice (Figure S9A–E, Supporting Information). Although we cannot exclude the possibility that besides NMDAR and mGluR5, other glutamate receptors may also participate in vHPC astrocytes‐mediated anxiety levels, our data strongly suggest that NMDAR plays an important role in mediating anxiety‐like behavior induced by vHPC astrocytic activation.

In the hippocampus, besides neurons, NMDAR has also been reported to be expressed in glial cells.^[^ ^17^ ^]^ To determine whether vHPC astrocytic activation produced anxiogenic effects through the activation of the NMDAR in local neurons, we used ifenprodil to selectively block the NMDA receptor subunit GluN2B, which is mainly located on extrasynaptic membranes, mediates the tonic NMDAR currents, and is tightly regulated by astrocytes.^[^ ^18^ ^]^ Furthermore, GluN2B receptor is present in neurons but not glial cells in hippocampal CA1.^[^ ^19^ ^]^ We found that intra‐vHPC application of ifenprodil (50 × 10^−6^ m) significantly improved anxiety‐like behaviors in vHPC astrocytes‐activated mice (Figure S10A–E, Supporting Information).

Together, these results show that an increase in glutamate levels in the vHPC accounts for the anxiogenic effect of astrocytic activation, as blockade of NMDAR with the NMDAR antagonists APV, memantine, HA966 or ifenprodil in the vHPC restored the enhanced anxiety‐like behaviors.

### 2.5 GFAP Expression and Astrocytic Activity were Increased in the vHPC After SubAcute Restraint Stress (SRS)

**Figure 5:**
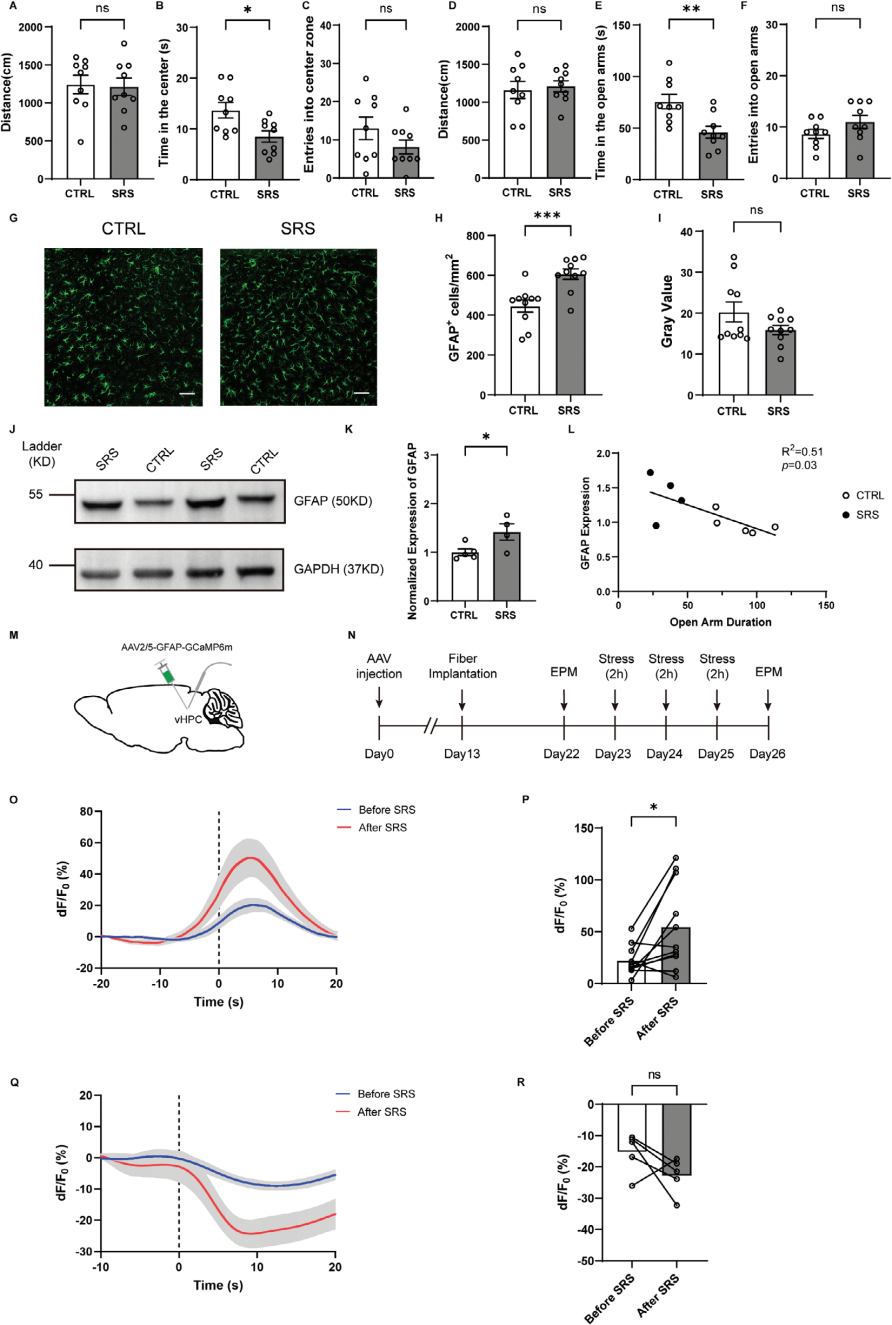
GFAP expression and astrocytic activity were increased in the vHPC after subacute restraint stress (SRS). A–F) SRS mice (*n* = 9) showed anxiety‐like behaviors in the OFT (A–C) and EPM (D–F) after SRS compared with control mice (CTRL, *n* = 9). G) Fluorescence images illustrating GFAP expression in the vHPC after SRS. Scale bar, 100 µm. H) The density of astrocytes was increased in the vHPC of SRS mice. I) The gray value of astrocytes was unaltered between groups. J,K) Western blot analysis showing increased GFAP levels in SRS mice relative to controls. L) Pearson correlation analysis of the time in open arms with the GFAP expression in the vHPC after SRS (*n* = 5, 4, *p* = 0.03, *R* ^2^ = 0.51). M) Schematic image showing AAV2/5‐GFAP‐GCaMP6m injection and fiber implantation in the vHPC. N) Experimental timeline for fiber‐based calcium recordings of hippocampal astrocytes during the EPM. O) Representative Ca^2+^ traces during open arm exploration in the EPM. The dotted line represents the time mice entered the open arms. Thick colored lines indicate mean and shaded areas indicate SEM. P) Calcium signal of vHPC astrocytes was increased during open‐arm exploration after SRS. *n* = 11 mice each group. Q) Same as (O) but during closed arm exploration. R) Same as (P) but during closed arm exploration. *n* = 5 mice each group. Data are presented as mean ± SEM; two‐tailed unpaired *t*‐test (A–F, H, I, K). Pearson correlation test (L). Two‐tailed paired *t*‐test (P, R). *P < 0.05, **P < 0.01, ***P < 0.001, ns, no significance. Each data point represents an individual mouse.

To determine whether vHPC astrocytes are associated with the pathological process of anxiety‐like behaviors, we first examined GFAP expression in the hippocampus of mice subjected to 3‐d SRS, a widely used mouse model of anxiety disorders.^[^ ^20^ ^]^ Mice were restrained for 2 h per day on 3 consecutive days and then subjected to anxiety‐like behavioral tests. SRS increased avoidance of OFT center zone (**Figure** **5**A–C) and EPM open arms (Figure 5D–F) compared with stress‐naive controls. These data demonstrate that mice exhibited enhanced anxiety‐like behaviors following exposure to 3‐d SRS.

Immunohistochemical staining for GFAP revealed a significant increase in the density of GFAP‐positive cells in the vHPC after SRS (Figure 5G,H), suggesting gliosis. Similar results have also been reported in rats displaying increased anxiety‐like behaviors following three‐week chronic restraint stress.^[^ ^21^ ^]^ However, no difference was observed for the mean gray value of GFAP‐positive cells (Figure 5I). Furthermore, Western blot analysis showed that GFAP protein expression level was significantly increased in SRS mice (Figure 5J,K). Together, these data suggest that the increase in GFAP level induced by SRS was more likely due to an increased astrocyte density than changes in GFAP expression in individual astrocytes. Correlation analysis showed that the time spent in EPM open arms was negatively correlated with GFAP protein level in the vHPC (Figure 5L). In addition, we tested cFos expression in vHPC neurons 30 min and 24 h after SRS and found that the number of cFos‐positive neurons in the vHPC exhibited a tendency to increase 30 min after SRS (Figure S11A,B, Supporting Information), whereas no change in neuronal activity was observed 24 h after SRS (Figure S11C,D, Supporting Information), suggesting that increased neuronal activity in the vHPC may underlie the enhanced anxiety‐like behaviors after SRS, which awaits further studies.

To investigate the impact of SRS on astrocytic Ca^2+^ level, we performed in vivo fiber photometry calcium imaging in mice subjected to SRS (Figure 5M). Three weeks after delivery of AAV2/5‐GFAP‐GCaMP6m in the vHPC, Ca^2+^ signals were recorded during EPM and OFT exploration before and after 3‐d SRS (Figure 5N). We found that vHPC astrocytes exhibited a significant increase in astrocytic Ca^2+^ activity during exploration of the anxiogenic open‐arm compartment in the EPM (Figure 5O–R) but not during center exploration in the OFT after SRS (Figure S1D, Supporting Information). The fact that the EPM is more anxiogenic than the OFT may underlie this inconsistency between the OFT and EPM tests.^[^ ^22^ ^]^ Together, these findings suggest that vHPC astrocytes are involved in the pathological process of anxiety‐like behaviors.

### 2.6 Genetic Inhibition of vHPC Astrocytes Conferred Resistance Against SRS, Whereas Chemogenetic Activation of vHPC Astrocytes Increased Susceptibility to SRS, Which Was Blocked by the NMDA Receptor Antagonist Memantine

**Figure 6:**
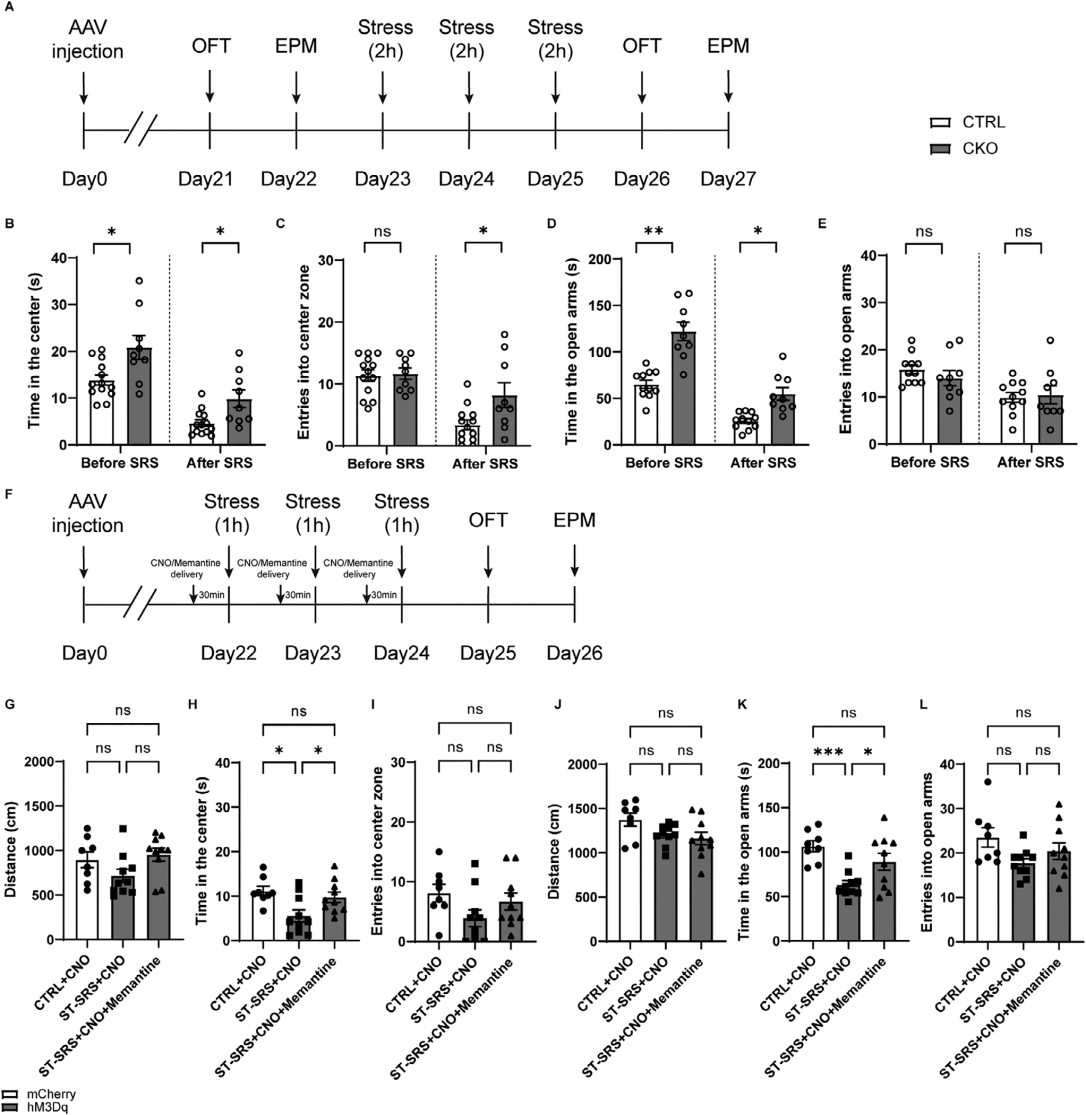
Genetic inhibition of vHPC astrocytes conferred resistance against SRS, whereas chemogenetic activation of vHPC astrocytes increased susceptibility to SRS, which was blocked by the NMDAR antagonist memantine. A) Experimental timeline. B–E) Astrocytic *Itpr2* knockout in the vHPC ameliorated anxiety‐like behaviors in the OFT (B, C, *n* = 9, 13) and EPM (D, E, *n* = 9, 11) in SRS animals. F) Experimental timeline. G–L) Chemogenetic activation of vHPC astrocytes induced anxiety‐like behaviors in the OFT (G–H, *n* = 8, 10, 10) and EPM (J–L) in mice subjected to subthreshold SRS (ST‐SRS), which were reversed by systemic application of the NMDA receptor antagonist memantine (10 mg kg^−1^, i.p.). Data are presented as mean ± SEM. Two‐way ANOVA followed by the Bonferroni post hoc test (B–E); one‐way ANOVA followed by the Bonferroni post hoc test (G–L). *P < 0.05, **P < 0.01, ***P < 0.001, ns, no significance. Each data point represents an individual mouse.

Given that conditional *Itpr2* knockout in vHPC astrocytes induced anxiolytic effects in stress naive mice, we next determined whether *Itpr2* deletion in vHPC astrocytes could confer resistance against the effects of SRS. Mice with *Itpr2* deletion in vHPC astrocytes were subjected to SRS and anxiety‐like behavioral tests were done before and after SRS (**Figure** **6**A). Anxiety‐like behaviors were not altered when mice were retested at a 3‐d interval (Figure S12, Supporting Information). We found that compared with control mice, CKO mice exhibited anxiolytic behaviors both before and after SRS as they spent more time in OFT center zone (Figure 6B,C) and EPM open arms (Figure 6D,E). Notably, CKO mice subjected to SRS exhibited reduced anxiety‐like behaviors to an extent similar to those in stress‐naive control mice (Figure 6B,D). These data suggest that genetic inhibition of vHPC astrocytic activity by astrocyte‐specific *Itpr2* knockout normalized anxiety‐like behaviors induced by SRS.

A recent study shows that acute optogenetic activation of astrocytes in the basolateral amygdala (BLA) can partially rescue chronic stress‐induced anxiety‐like behaviors.^[^ ^23^ ^]^ We next determined whether acute chemogenetic activation of vHPC astrocytes has any effect on the enhanced anxiety‐related behaviors in SRS mice (Figure S13A, Supporting Information). As expected, compared to mCherry mice, hM3Dq mice displayed enhanced anxiety‐related behaviors in stress‐naive conditions (Figure S13B–E, Supporting Information). Notably, when both mCherry mice and hM3Dq mice were subjected to SRS, acute chemogenetic activation of vHPC astrocytes worsened anxiety‐like behaviors as evidenced by a further decrease in exploration time and entry number in OFT center zone (Figure S13B,C, Supporting Information) and EPM open arms (Figure S13D,E, Supporting Information). Thus, in marked contrast to BLA astrocyte activation,^[^ ^23^ ^]^ acute chemogenetic activation of vHPC astrocytes deteriorated anxiety‐like behaviors in SRS mice.

Given that chemogenetic activation of vHPC astrocytes was anxiogenic in stress naive mice and vHPC astrocytic activity was increased after SRS, we then tested whether chemogenetic activation of vHPC astrocytes increased susceptibility to SRS. Mice subjected to subthreshold SRS (ST‐SRS, 1‐hour restraint per day for three consecutive days) did not display anxiety‐like behaviors (Figure S14, Supporting Information). However, when ST‐SRS was paired with chemogenetic activation of vHPC astrocytes (Figure 6F), anxiety‐like behaviors were enhanced in the OFT(Figure 6G–I) and EPM (Figure 6J–L). We went on to test whether memantine prevents the induction of anxiety‐like behaviors in mice subjected to treatment of both ST‐SRS and chemogenetic activation of vHPC astrocytes. Memantine (10 mg kg^−1^, i.p.) was co‐applied with CNO 30 min prior to ST‐SRS (Figure 6F). The concentration of memantine was chosen based on previous reports.^[^ ^24^ ^]^ Surprisingly, we found that memantine administration fully blocked the anxiety‐like behaviors induced by co‐treatment of ST‐SRS and chemogenetic activation of vHPC astrocytes (Figure 6G–L).

Taken together, our findings demonstrate that in both stress‐naive and stressed mice, genetic inhibition of vHPC astrocytes was anxiolytic and increased resistance against SRS, whereas chemogenetic activation of vHPC astrocytes was anxiogenic and increased susceptibility to SRS, supporting the hypothesis that vHPC astrocytes bidirectionally regulated innate and pathological anxiety‐like behaviors.

### 2.7 Blockade of NMDAR in the vHPC Attenuated the Anxiogenic Effects Induced by SRS

**Figure 7:**
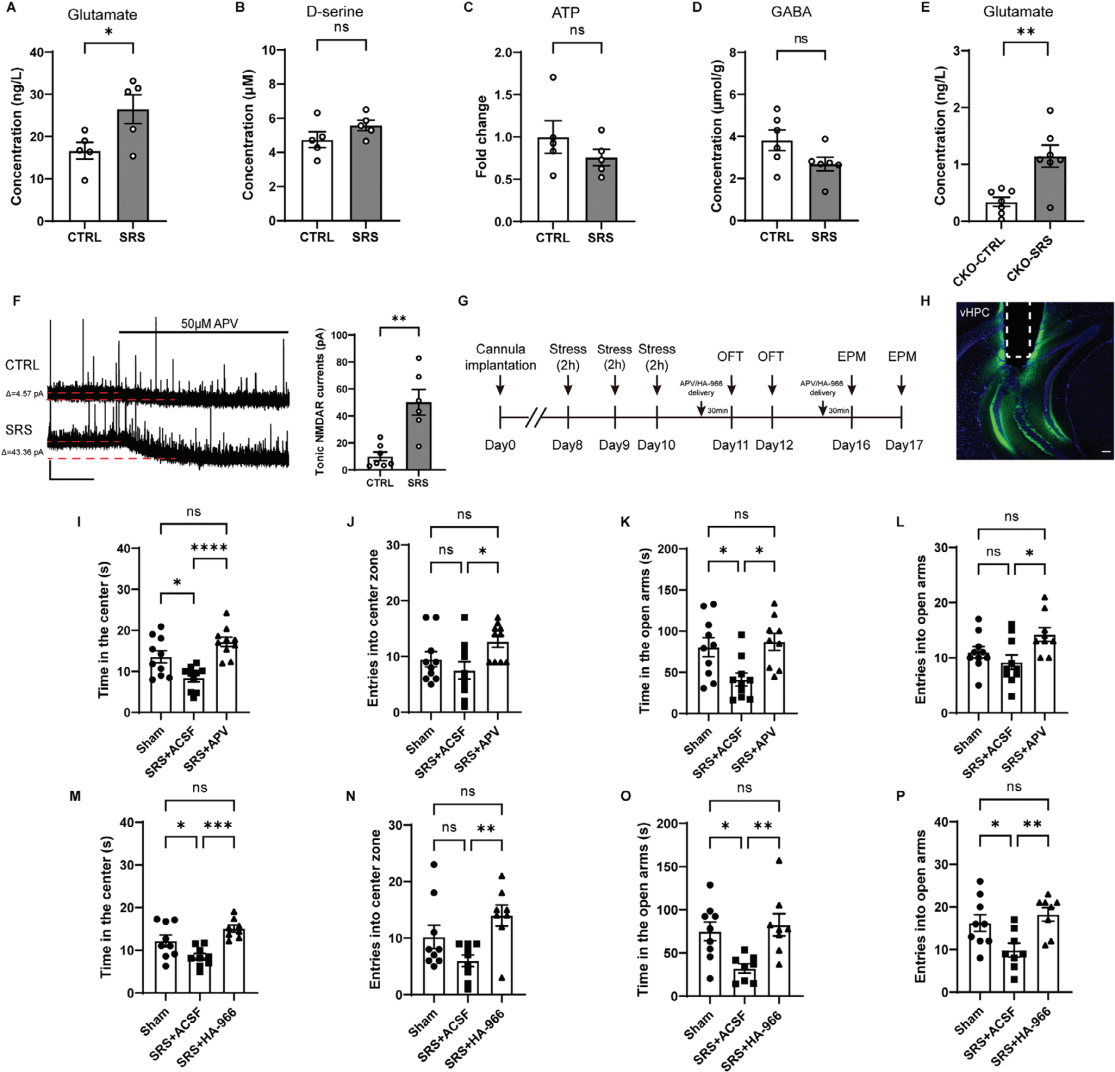
Intra‐vHPC application of APV or HA‐966 rescued SRS‐induced anxiety‐like behaviors. A–D) The levels of glutamate, d‐serine, ATP, and GABA from SRS and control mice. *n* = 5 (A–C) or 6 (D) per group. E) The levels of glutamate in CKO mice with or without SRS. *n* = 7 per group. F) Sample traces of tonic NMDAR current recordings from vHPC CA1 pyramidal neurons in control and SRS mice (left) and quantification of tonic NMDAR currents (right, *n* = 7, 6 cells from 6 mice). Black lines indicate the changes of baseline induced by application of APV (50 × 10^−6^ m). Scale bars: 2 min, 50 pA. G) Experimental timeline. H) Confocal image showing the placement of the cannula above the vHPC and the expression of CTB488 mixed with APV in the vHPC. Scale bar, 100 µm. I–L) 5 × 10^−3^ m APV rescued anxiety‐like behaviors in the OFT (I, J, *n* = 10, 10, 10) and EPM (K, L, *n* = 10, 10, 9) in SRS animals. M–P) Local application of (R)‐(+)‐HA‐966 (50 × 10^−3^ m) rescued anxiety‐like effects in the OFT (M, N, *n* = 9, 9, 8) and EPM (O, P, *n* = 9, 8, 8) in SRS mice. Data are presented as mean ± SEM; two‐tailed unpaired *t* test (A–E); one‐way ANOVA followed by the Bonferroni post hoc test (I–P). *P < 0.05, **P < 0.01, ***P < 0.001, ****P < 0.0001, ns, no significance. Each data point represents an individual mouse (A–E, I–P).

To determine whether gliotransmitters are involved in SRS‐induced anxiety‐like behaviors, we first measured the levels of glutamate, GABA, ATP, and d‐serine in vHPC slices collected from controls and SRS mice. Surprisingly, we found that as in stress‐naive hM3Dq mice, only the level of glutamate but not GABA, ATP, or d‐serine was increased in hippocampal slices of SRS mice (**Figure** **7**A–D). To determine if SRS led to an increase in ambient glutamate levels in an astrocytic IP3R2‐dependent manner, glutamate levels in the vHPC of CKO mice with or without SRS were examined. Surprisingly, we still found a significant increase in glutamate levels in stressed CKO mice compared to their stress‐naive counterparts (Figure 7E). The remaining astrocytes without IP3R2 CKO as well as other cells including neurons or microglia may contribute to this glutamate increase after SRS. Of note, the baseline glutamate levels in stress‐naive CKO mice were far lower than that in stress‐naive control mice with or without vHPC astrocytic activation (Figure 7E vs Figures 4A and 7A), suggesting that astrocytes play a critical role in maintaining the baseline ambient glutamate levels in an IP3R2‐dependent manner, consistent with the notion of astrocytes as a major origin of ambient glutamate.^[^ ^25^ ^]^ To determine whether ambient glutamate increase in SRS mice led to an increase in tonic NMDAR currents in adjacent neurons, we recorded vHPC CA1 pyramidal neurons at a holding potential of +40 mV in the presence of the NMDAR antagonist APV (50 × 10^−6^ m) (Figure 7F). The tonic NMDAR currents were significantly increased in vHPC CA1 pyramidal neurons in SRS mice compared to stress‐naive controls (Figure 7F), confirming a glutamate excess in the vHPC of SRS mice.

We then tested whether glutamate excess contributes to the enhanced anxiety‐like behaviors in SRS mice (Figure 7G). CTB488 mixed with APV was delivered to the vHPC to show the site of cannula implantation (Figure 7H). We found that intra‐vHPC APV (5 × 10^−3^ m) application fully rescued the enhanced anxiety‐like behaviors in SRS mice (Figure 7I–L), as no difference was observed between APV‐treated SRS mice and sham controls. The APV‐mediated rescuing effect on anxiety‐like behaviors was transient as it disappeared 24 h after administration (Figure S15, Supporting Information). Similar anxiolytic effects were observed when the GluN2B‐specific antagonist ifenprodil (50 × 10^−6^ m) but not the mGluR5 antagonist MPEP (20 × 10^−6^ m) was locally delivered to the vHPC in SRS mice (Figures S9F–L and S10F–L, Supporting Information). We also tested the effect of (R)‐(+)‐HA‐966 (50 × 10^−3^ m) on the enhanced anxiety‐like behaviors caused by SRS. As expected, application of HA966 (50 × 10^−3^ m) in the vHPC induced transient anxiolytic effects and rescued the anxiety phenotypes in SRS mice (Figure 7M–P and Figure S16, Supporting Information). Together, these results show that an increase in glutamate level in the vHPC accounts for the anxiogenic effect of SRS, as blockade of NMDAR in the vHPC restored the enhanced anxiety‐like behaviors caused by SRS.

### 2.8 Systemic or Local Application of Memantine Fully Rescued SRS‐Induced Anxiety‐Like Behaviors

**Figure 8:**
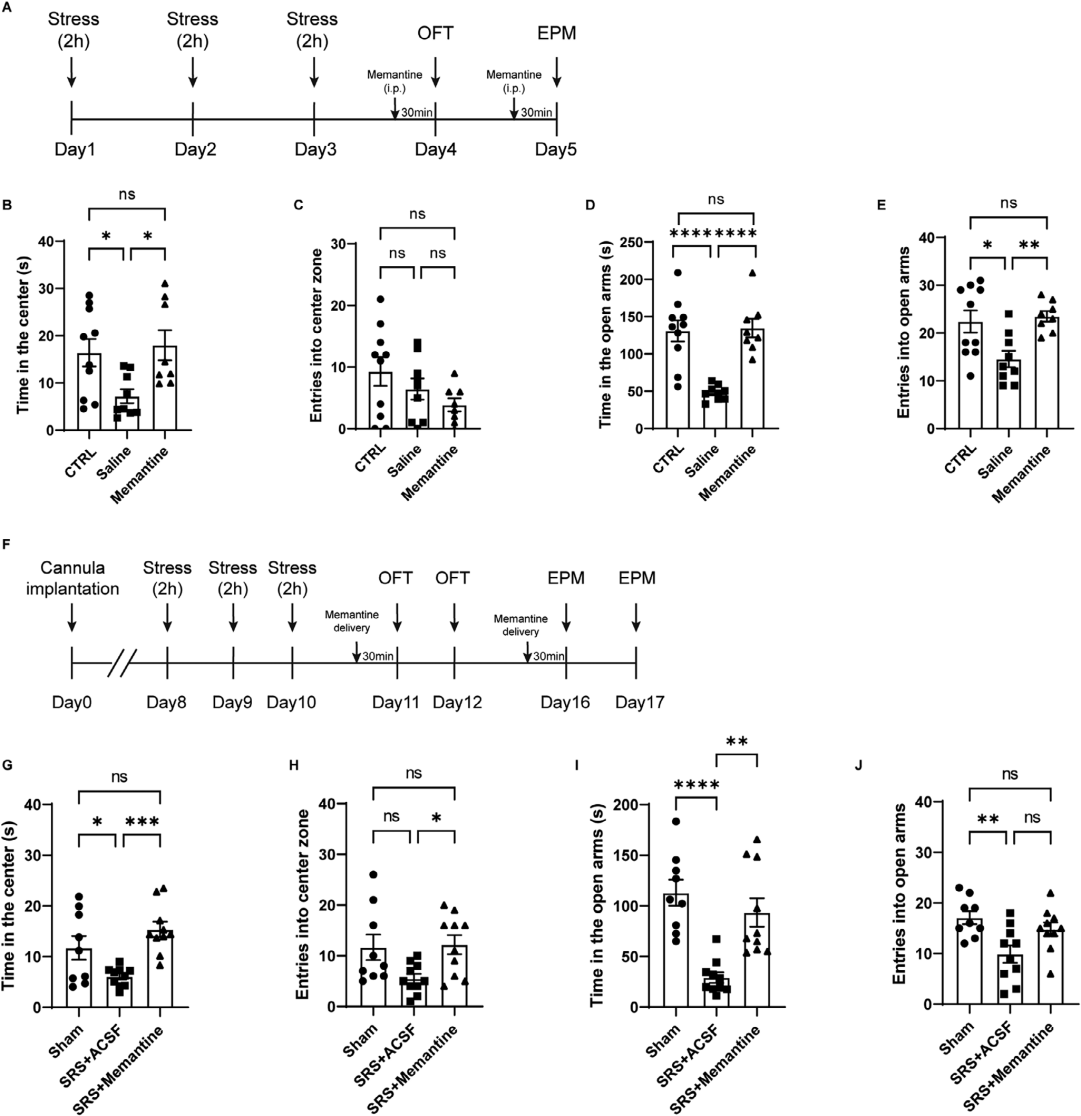
Systemic or local application of memantine rescued anxiety‐like behaviors in SRS mice. A) Experimental timeline. B–E) Systemic application of memantine (10 mg kg^−1^, i.p.) rescued anxiety‐like behaviors in the OFT (B, C, *n* = 10, 9, 8) and EPM (D, E, *n* = 10, 9, 8) in SRS animals. F) Experimental timeline. G–J) Same as (B)–(E) but for local delivery of memantine (50 × 10^−3^ m) in the vHPC in the OFT (G, H, *n* = 9, 10, 10) and EPM (I, J). Data are presented as mean ± SEM; one‐way ANOVA followed by the Bonferroni post hoc test (B–E, G–J). *P < 0.05, **P < 0.01, ***P < 0.001, ****P < 0.0001, ns, no significance. Each data point represents an individual mouse (B–E, G–J).

We then tested whether memantine exerts anxiolytic effects on SRS‐induced anxiety‐like behaviors. A single i.p. injection of memantine at a dose of 10 mg kg^−1^ was applied 30 min prior to behavioral tests in SRS mice (**Figure** **8**A). As expected, the enhanced anxiety‐like behaviors in SRS mice were fully rescued by a single systemic application of memantine (Figure 8B–E).

To determine whether memantine produced anxiolytic effects in SRS mice through vHPC, behavioral tests were conducted after intra‐vHPC administration of memantine. We found that local delivery of memantine (50 × 10^−3^ m) produced transient anxiolytic effects in stressed mice (Figure 8F–J and Figure S17, Supporting Information), highlighting a critical role for the vHPC in mediating the anxiolytic effects of memantine.

### 2.9 Adenosine A1 Receptor (A1R) Did Not Mediate the Anxiety‐Like Behaviors Induced by Astrocytic Activation or SRS

A recent study showed that optogenetic activation of dHPC astrocytes reduces fear‐related anxiety‐like behaviors through A1R.^[^ ^26^ ^]^ To determine whether A1R is involved in anxiety‐like behaviors induced by chemogenetic activation of vHPC astrocytes or by SRS, 8‐cyclopentyl‐1,3‐dimethylxanthine (CPT), a specific A1R antagonist was injected into the vHPC after astrocytic activation (Figure S18G, Supporting Information) or SRS (Figure S18L, Supporting Information). We selected CPT (1 × 10^−3^ m) for subsequent experiments as it had no effect on baseline anxiety‐related behaviors in wild‐type mice after local application to the vHPC (Figure S18A–F, Supporting Information). Local delivery of CPT (1 × 10^−3^ m) to the vHPC exerted no rescuing effect on the enhanced anxiety‐related behaviors in astrocyte‐activated mice (Figure S18H–K, Supporting Information) or in SRS mice 30 min (Figure S18M–P, Supporting Information) or 24 h after delivery (Figure S18Q–T, Supporting Information). Together, these results indicate that A1R was not involved in anxiety‐like behaviors induced by astrocytic activation or by SRS.

## 3 Discussion

In this study, we provided multiple lines of evidence supporting the notion that vHPC astrocytes can bidirectionally regulate innate and stress‐induced anxiety‐like behaviors. First, vHPC astrocytic Ca^2+^ activity was increased by anxiogenic stimuli and by subacute stress. Second, genetic inhibition of vHPC astrocytes was anxiolytic, whereas chemogenetic or stress‐induced activation of vHPC astrocytes was anxiogenic under both normal and pathological conditions. Third, extracellular glutamate level was increased in the vHPC following chemogenetic or stress‐induced activation of vHPC astrocytes. Fourth, intra‐vHPC application of the NMDAR antagonists APV, ifenprodil, memantine, or HA966 normalized anxiety‐like phenotypes in astrocyte‐activated stress‐naive mice and in stressed mice. Fifth, chemogenetic activation of vHPC astrocytes increased susceptibility to SRS, which was prevented by systemic application of memantine. Sixth, a single i.p. application of memantine fully rescued SRS‐induced anxiety‐like behaviors.

We found that Gq‐DREADD‐mediated chemogenetic activation of vHPC astrocytes produced anxiogenic effects. This is in striking contrast to a recent study showing that ChR2‐mediated optogenetic stimulation of hippocampal astrocytes induces anxiolytic effects regardless of the structural and functional heterogeneity of the hippocampus along the dorsoventral axis.^[^ ^5^, ^27^ ^]^ Optogenetic activation of dHPC or vHPC astrocytes leads to activation of the whole hippocampus, manifested as an increase in cFos expression.^[^ ^5^ ^]^ Due to the subregion‐ and projection‐specific regulation of anxiety‐related behaviors, with some vHPC projections producing opposing effects,^[^ ^4a–f^ ^]^ this widespread activation of the hippocampus induced by optogenetic activation of dHPC or vHPC astrocytes may mask some of the specific effects of subregion‐specific astrocyte activation on hippocampal neuronal activity in normal and stress conditions. Although astrocytes are initially considered as a homogenous population, mounting evidence indicates that in the adult hippocampus, astrocytes exhibit inter‐ and intra‐subregional functional heterogeneity, differentially respond to neuronal activity changes and regulate neuronal activity and behaviors in a subregion‐ and projection‐dependent manner.^[^ ^28^ ^]^ For example, vHPC astrocytes play an important role in resistance to stress,^[^ ^7^ ^]^ whereas dHPC astrocytes are essential for learning and spatial memory.^[^ ^12^, ^28^ ^]^ Furthermore, Gi‐DREADD‐mediated chemogenetic activation of dHPC astrocytes impairs remote memory formation by projection‐specific inhibition of anterior cingulate cortex‐projecting, but not nucleus accumbens‐projecting, dHPC neurons.^[^ ^28b^ ^]^ The principle of projection‐specific response and functions of astrocytes may apply to astrocytes in other brain regions such as the mPFC and amygdala.^[^ ^29^ ^]^

In contrast to a widespread cFos expression in the hippocampus by optogenetic activation of dHPC or vHPC astrocytes,^[^ ^5^ ^]^ we found that Gq‐DREADD‐mediated chemogenetic activation of vHPC astrocytes induced cFos expression in hM3Dq‐mCherry‐expressing astrocytes and neighboring neurons in the vHPC but not dHPC. The reasons for the difference may lie in at least two aspects. First, previous studies have showed that ChR2‐mediated excitation of astrocytes is sufficient to enhance neuronal excitability by increasing extracellular K^+^ concentration.^[^ ^30^ ^]^ In contrast, Gq‐GPCR‐mediated astrocytic Ca^2+^ signals decrease extracellular K^+^ concentration,^[^ ^31^ ^]^ an important physiological function of astrocytes in maintaining ion homeostasis. Second, the presence of fiber‐optic cables within the hippocampus may lead to injury that causes activation of reactive astrocytes and microglia during optogenetic manipulation. However, chemogenetics poses the advantage of being less invasive as the designer synthetic ligand CNO can be simply delivered by systemic injection that causes minimal disturbance in behaving animals.

Optogenetic and chemogenetic approaches have been widely used to study the functional roles of astrocytes in the regulation of anxiety. For example, optogenetic stimulation of astrocytes in the centrolateral amygdala (CeL) increases CeL neuronal activity possibly by releasing d‐serine, which, in turn, inhibits neurons in the centromedial amygdala (CeM), resulting in anxiolysis in rats with neuropathic pain.^[^ ^32^ ^]^ Optogenetic activation of dHPC astrocytes reduces fear‐related anxiety‐like behaviors through A1R in rats.^[^ ^26^ ^]^ Chronic (21 d) optogenetic stimulation of astrocytes in the BLA can fully rescue chronic stress‐induced anxiety‐like phenotypes in mice.^[^ ^23^ ^]^ Notably, acute optogenetic activation of astrocytes in the CeL and BLA produces little effect on innate anxiety‐like behaviors.^[^ ^32^, ^33^ ^]^ Similarly, both photostimulation of dHPC astrocytes and inhibition of dHPC astrocyte Ca^2+^ signaling by expression of the Ca^2+^‐extruder PMCA2w/b in dHPC astrocytes also have no effect on innate anxiety‐like behaviors.^[^ ^26^ ^]^ From these above studies, it seems that acute optogenetic activation of astrocytes in brain regions essential for stress response has marginal effect on innate anxiety, whereas in our study, we found that acute chemogenetic activation of vHPC astrocytes increased innate and stress‐induced anxiety. There are multiple reasons for this difference, and one worth noting is that optogenetic and chemogenetic activation of astrocytes may lead to the release of different gliotransmitters through distinct mechanisms. In support of this notion, acute optogenetic activation of dHPC astrocytes leads to selective release of ATP/adenosine,^[^ ^5^, ^26^ ^]^ whereas in this study, we found that acute chemogenetic activation of vHPC astrocytes resulted in a selective increase of extracellular glutamate level. Selective astrocyte glutamate release initiated by Gq‐DREADD has also been reported in the nucleus accumbens core to inhibit cue‐induced cocaine seeking.^[^ ^34^ ^]^ Although the mechanisms underlying gliotransmission induced by optogenetic or chemogenetic activation of astrocytes remain to be elucidated,^[^ ^35^ ^]^ previous studies have shown that ChR2‐mediated stimulation of astrocytes bypasses endogenous GPCR‐IP3R‐Ca^2+^ signaling cascade,^[^ ^36^ ^]^ and the release of gliotransmitters caused by ChR2‐mediated stimulation of astrocytes may reflect an astrocytic acidification that can lead to gliotransmission, as activation of ChR2 increases intracellular H^+^ level and astrocytic alkalization leads to cessation of gliotransmission.^[^ ^35^, ^36^, ^37^ ^]^ In contrast, Gq‐DREADD activation of astrocytes leads to the release of gliotransmitters through IP3R2‐mediated Ca^2+^ signaling pathway, similar to endogenous Gq‐GPCR activation.^[^ ^13^, ^29^ ^]^ Notably, in contrast to optogenetic activation, Gq‐GPCR‐coupled oxytocin signaling in CeL astrocytes is essential for modulation of innate anxiety‐like behaviors, as deletion of oxytocin receptors in these cells not only abolishes the anxiolytic effects of oxytocin but also increases innate anxiety‐like behaviors.^[^ ^32^ ^]^

Chronic stress can lead to maladaptive alterations in glutamatergic function in the frontal limbic cortex. Neuroimaging and post‐mortem studies have demonstrated increased glutamate levels in the frontal cortex in patients with MAD.^[^ ^38^ ^]^ Both acute and chronic stress increases extracellular glutamate levels in the hippocampus in rodents,^[^ ^39^ ^]^ which may contribute to a loss of stress resilience and hippocampal dysfunction in MAD.^[^ ^7^, ^40^ ^]^ In line with these findings, in this study, we found that Gq‐DREADD‐mediated acute activation of vHPC astrocytes and SRS increased intra‐astrocytic Ca^2+^ levels and extracellular glutamate levels,^[^ ^41^ ^]^ and vHPC astrocytic modulation of innate and stress‐induced anxiety‐like behaviors involved NMDAR, supporting an excess of glutamate as an underlying mechanism. In support of this notion, previous studies have showed that Gq‐DREADD‐mediated activation of hippocampal astrocytes increases intracellular Ca^2+^ levels leading to the release of glutamate.^[^ ^13a^ ^]^ Notably, increasing the extracellular glutamate levels by blockade of glutamate uptake in the central amygdala is sufficient to induce anxiety‐ and depressive‐like behaviors.^[^ ^42^ ^]^ Although the glutamate level in the vHPC was selectively increased after acute chemogenetic activation of vHPC astrocytes and after SRS, the possibility cannot be excluded that other neurotransmitters or neuromodulators may also be involved in regulating innate and pathological anxiety in vHPC astrocyte‐activated mice and in stressed mice. Of note, we found that the levels of the major inhibitory neurotransmitter GABA were largely unaltered in the vHPC in stressed mice, which differs from previous findings of a GABA deficit as a hallmark of anxiety disorders.^[^ ^43^ ^]^ The mechanisms underlying this difference are currently unknown, and one potential reason is that a subacute stress model was used in this study, whereas patients with anxiety disorders tend to suffer from chronic stress, which may lead to a GABA deficit, although more future works are required to test this hypothesis. Besides, several questions remain unanswered and await further studies. For example, are astrocytes the only source of excessive extracellular glutamate? What are the mechanisms underlying the glutamate excess? Is it due to exaggerated release or failed clearance or a combination of both? Are the vHPC neurons affected by chemogenetic activation of vHPC astrocytes or by SRS projection‐specific, as previously reported?^[^ ^28^, ^29^ ^]^ Are these vHPC neurons subregion‐specific? That is, do vHPC CA1 or CA3 or DG neurons mediate the effects of astrocyte manipulation on anxiety?

## 4 Conclusion

In summary, our findings identify novel roles and mechanisms for vHPC astrocytes in bidirectionally regulating physiological and pathological anxiety‐like behaviors, highlighting vHPC astrocytes as potential therapeutic targets for anxiety and anxiety‐related disorders.

## 5 Experimental Section

### Animals

Adult male C57BL/6J mice aged 8–10 weeks were obtained from the Laboratory Animal Center at Southern Medical University in Guangzhou, China. Mice were housed in standard laboratory cages (four to five per cage) on a 12 h light/dark cycle (lights on at 8:00 a.m.) in a temperature‐controlled room (21–25 °C). Food and water were given ad libitum.

IP3R2^loxp/loxp^ mice were a kind gift from Dr. Ju Chen. The genotyping primers for IP3R2^loxp/loxp^ mice PCR were 5′‐GCTGTGCCCAAAATCCTAGCACTG‐3′ and 3′‐CATGCAGAGGTCGTGTCAGTCATT‐5′.

At 8 weeks of age, mice were injected with virus and drugs as part of the experimental procedure. All procedures were conducted in accordance with the Guidelines of the Chinese Council on Animal Care and approved by the Institutional Animal Care and Use Committee (IACUC) of Southern Medical University (L‐2016‐046, Guangdong, China). Efforts were made to minimize the suffering of the animals and to reduce the number of animals used in the study.

### Virus Injection and Fiber/Guide Cannula Implantation

All surgeries were performed under aseptic conditions under stereotaxic guidance. Mice were anesthetized using 0.75% pentobarbital sodium and placed into a stereotaxic instrument (RWD, China). All coordinates are reported in mm relative to bregma. Injections were performed with a micropipette connected to a Nanoliter Injector (Nanoliter 2010, WPI, Sarasota, FL) and its controller (Micro4, WPI, Sarasota, FL) at an 80 nL min^−1^ rate. The micropipette was then slowly retracted after 10 min. Only mice with virus expression restricted to the target region were used.

To conditionally knock out *Itpr2* in vHPC astrocytes, AAV2/5‐gfaABC1D‐Cre‐EGFP or AAV2/5‐gfaABC1D‐EGFP (SunBio, Shanghai, China) was bilaterally injected into the vHPC of IP3R2^loxp/loxp^ mice at the following coordinates: AP: −3.28, ML: ±3.25, and DV: −3.60 and −3.35 (230 nL/site). To conditionally knock out *Itpr2* in vHPC neurons, AAV2/9‐CamkII‐Cre‐EGFP or AAV2/9‐CamkII‐EGFP (BrainVTA, Wuhan, China) was bilaterally injected into the vHPC of IP3R2^loxp/loxp^ mice at the following coordinates: AP: −3.28, ML: ±3.25, and DV: −3.60 and −3.30 (230 nL/site).

To induce hM3Dq expression on vHPC astrocytes, AAV2/5‐GFAP‐hM3Dq‐mCherry or AAV2/5‐GFAP‐mCherry (WZ Biosciences, Jinan, China) was bilaterally injected into the vHPC (230 nL/site). The designer drug clozapine‐N‐oxide (CNO; 3 mg kg^−1^, i.p.; #HY17366, MedChemExpress) was administered 30 min before the behavioral tests.

For fiber photometry recording, AAV2/5‐GFAP‐GCaMP6m (SunBio, Shanghai, China) was unilaterally injected in the vHPC (coordinates: AP: −3.28, ML: ±3.25, and DV: −3.45, 280 nL/site). For fiber implantation surgery, mice labeled with GCaMP6m were anesthetized with pentobarbital sodium and placed in a stereotaxic head frame. An optical fiber (diameter of 200 µm, numerical aperture 0.37; Newdoon, China) was inserted into the location where the virus was injected for fiber placement (coordinates: AP: −3.28, ML: ±3.25, and DV: −3.40).

For Ca^2+^ imaging, AAV2/5‐GFAP‐hM3Dq‐mCherry mixed with AAV2/5‐GFAP‐GCaMP6m was bilaterally injected into the vHPC (230 nL/site) three weeks before slice preparation.

For mice used in drug injection experiments, a guide cannula was unilaterally implanted into the vHPC (AP: −3.28, ML: ±3.25, and DV: −3.40) of mice randomly. After a one‐week recovery, the behavioral tests were performed 30 min after infusion.

### Drug Delivery

For glutamate receptor antagonist infusion, the solution consisted of 50 × 10^−3^ m d(‐)−2‐amino‐5‐phosphonopentanoic‐acid (APV; #A8054, Sigma‐Aldrich) was dissolved in ACSF and stored at −20 °C. To prepare the working solution for experiments, stock solution of APV was diluted with ACSF at the dose of 5 × 10^−3^ m before use. The solution consisted of 50 × 10^−3^ m (R)‐(+)‐HA‐966 (HA966; #0281, Tocris) was dissolved in ACSF and stored at −20 °C. Ifenprodil hemitartrate (#ab120111, Abcam), a GluN2B‐preferring NMDAR antagonist, was dissolved in PBS with 5% DMSO and 9% Tween80 and injected intrahippocampally (50 × 10^−6^ m) 30 min before behavioral tests. The solution consisted of 20 × 10^−6^ m MPEP hydrochloride (#1212, Tocris), a metabotropic glutamate receptor 5 antagonist, was dissolved in ACSF and prepared before use. For A1 adenosine receptor antagonist infusion, the solution consisted of 1 × 10^−3^ m 8‐cyclopentyl‐1,3‐dimethylxanthine (CPT; #C102, Sigma‐Aldrich) was dissolved in ACSF before use. Thirty minutes before the behavioral assays, 0.3 µL of solution was infused via infusion cannulae at a flow rate of 0.1 µL min^−1^. The infusion cannulae (62203, RWD, China) were connected via polyethylene tubing (62302, RWD, China) to 10 µL microsyringes (Hamilton, Reno, NV) mounted on a microinfusion pump (RWD200, China). To allow for the diffusion of the drug, the infusion cannulae were kept in place for 5 min before being replaced with dummy cannulae. Memantine (#M9292, Sigma‐Aldrich), an NMDA receptor antagonist, was dissolved in saline and injected intraperitoneally (10 mg kg^−1^) or intrahippocampally (50 × 10^−3^ m) 30 min before behavioral tests.

### Histology and Microscopy

Mice were anesthetized using intraperitoneal injection of pentobarbital sodium, followed by transcardial perfusion with 0.9% saline and 4% paraformaldehyde (PFA) in PBS. After careful removal of the brains, the brains were postfixed in 4% PFA at 4 °C overnight and subsequently transferred to 30% sucrose. Coronal slices measuring 40 µm in thickness were obtained from the brain tissue using a freezing microtome (Leica, CM1950) following immersion in sucrose solution. Finally, the tissue sections were visualized using a laser confocal microscope (Nikon C2, Japan).

For immunocytochemistry, sections were washed with PBS three times and were then blocked in 5% normal goat serum solution containing 0.1% Triton X‐100 for 2 h at room temperature. Then, slices were incubated with primary antibody (anti‐GFAP, 1:500, #3670S, Cell Signaling Technology; anti‐cFos, 1:800, #ABE457, Millipore; anti‐NeuN, 1:1000, #ab177487, abcam) at 4 °C overnight and washed three times in PBS before incubation for 2 h at room temperature with Alexa Fluor‐conjugated secondary antibodies (1:1000, goat anti‐rabbit IgG (H+L) secondary antibody, Alexa Fluor 488 conjugate, #A‐11008; goat anti‐mouse IgG (H+L) secondary antibody, Alexa Fluor 647 conjugate, #A‐32728; goad anti‐rat IgG (H+L) secondary antibody, Alexa Fluor 594 conjugate, #A‐11007; Invitrogen). Quantitative analysis was performed with ImageJ software. Using ImageJ, color values of the pixels were converted to corresponding gray values, and the mean gray value was the sum of the gray values of all the pixels in the image divided by the number of pixels.

### Western Blot Analysis

Brain tissues were lysed in ice‐cold lysis buffer (#FD009, FudeBio) containing 1 mmol L^−1^ protease inhibitor (PMSF). The samples were then centrifuged for 30 min at 16 000×*g* at 4 °C, and the supernatant was collected and quantified with the Microplate BCA Protein Assay Kit (#23227, Thermo). The protein samples were separated by 10% SDS‐PAGE gels and transferred to PVDF membranes (Millipore). The membranes were blocked with 5% defatted milk at room temperature for 1 h and then incubated overnight with a primary antibody (anti‐GFAP, 1:1000, #3670S, Cell Signaling Technology) at 4 °C. Antibody binding was detected by incubation with an HRP‐conjugated secondary antibody (1:5000, #FDM007, FudeBio) at room temperature for 1 h. The protein expression levels were evaluated by quantifying the gray density of the western blot bands with AlphaEaseRFC software (Alpha Innotech Corporation). All samples were normalized to internal controls.

### Quantitative Real‐Time PCR

Brain samples were dissected and processed according to the previously described methods.^[^ ^44^ ^]^ Separate tissue samples were immediately stored in TRIZOL (Invitrogen), and RNA was extracted according to the manufacturer's instructions. Genomic DNA was removed using gDNA eraser treatment (TaKaRa), and 1 µg RNA was used for first‐strand cDNA synthesis (TaKaRa). For RT‐qPCR, a SYBR detection system (TaKaRa) and 2 µL of undiluted cDNA were used in 20 µL total volume PCRs. Each reaction was performed in duplicate. All real‐time (RT)‐PCRs were performed over 40 cycles using an iCycler (Agilent Technologies Stratagene Mx3005P). Relative gene expression and statistical analysis were determined using the Relative Expression Software Tool. The primers were designed and synthesized as follows: *Itpr2* sense 5′‐TGGTGGATGACCGTTGTG‐3′, antisense 5′‐GTATTGCTTCTGGGCAGAGTAT‐3′. The expression of target genes was normalized against the expression of 18S as an endogenous control gene.

### Slice Preparation

Male mice (aged 2–3 months) were anesthetized with pentobarbital and then decapitated. The brains were removed quickly and placed into ice‐cold cutting solution containing (in mm): 250 sucrose, 26 NaHCO_3_, 10 glucose, 10 MgSO_4_, 2 KCl, 1.3 NaH_2_PO_4_, and 0.2 CaCl_2_. Slices containing the vHPC (300 µm) were prepared in ice‐cold cutting solution using a VT‐1200S vibratome (Leica, Germany), transferred to the storage chamber containing regular ACSF (in mm: 126 NaCl, 26 NaHCO_3_, 10 glucose, 3 KCl, 2 CaCl_2_, 1.25 NaH_2_PO_4_, and 1 MgSO_4_), and allowed to recover at 34 °C for 30 min and then at room temperature (25 ± 1 °C) for 1 h before recording. During the slice preparation, all solutions were saturated with 95% O_2_/5% CO_2_ (vol/vol).

### Electrophysiological Recordings

Slices were placed in the recording chamber that was superfused (3 mL min^−1^) with ACSF at 32–34 °C. Whole‐cell patch‐clamp recordings of ventral CA1 pyramidal neurons were obtained under an infrared‐differential interference contrast microscope (ECLIPSE FN1, Nikon). For tonic NMDAR‐mediated current recording, neurons were held at +40 mV in ACSF with an internal solution containing (in mm): 130 Cs methylsulfate, 10 NaCl, 4 ATP‐Mg, 0.3 Na_3_‐GTP, 10 EGTA, 10 HEPES, pH adjusted to 7.4 with CsOH, osmolality 295 mOsm with sucrose. For tonic NMDAR current recording, after baseline was stable, tonic NMDAR current was observed by bath applying APV. Data were recorded with a multiClamp 700B (Molecular Devices), digitized at 5 kHz, and filtered at 1 kHz. Data were collected when the series resistance fluctuated within 20% of the initial values and analyzed using pClamp10.2 software (Molecular Devices).

### Fiber Photometry

Following AAV‐GFAP‐GCaMP6m virus injection, a ceramic ferrule with an optical fiber (diameter of 200 µm, numerical aperture (NA) 0.37; Newdoon, China) was implanted with the fiber tip in the vHPC through the craniotomy. The ceramic ferrule was stabilized with dental acrylic. Signals were recorded three weeks after virus injection and one week after optical fiber implantation. Calcium signal recording was performed as described.^[^ ^45^ ^]^ To record fluorescence signals, laser beam with a wavelength of 488 nm (OBIS 488LS; Coherent) was reflected using a dichroic mirror (MD498; Thorlabs) focused by a 10× objective lens (NA = 0.3; Olympus) and then coupled to an optical commutator (Doric Lenses). An optical fiber (200 mm O.D., NA = 0.37, 2‐m long) guided the light between the commutator and the implanted optical fiber. To minimize photobleaching, the laser power at the fiber tip was adjusted to 30 µW. The GCaMP fluorescence was filtered using a bandpass filter (MF525‐39, Thorlabs) and detected using a photomultiplier tube (R3896, Hamamatsu). The output current from the photomultiplier tube was converted to voltage signals using an amplifier (C7319, Hamamatsu). These signals were then filtered through a low‐pass filter (cut‐off at 40 Hz; Brownlee 440). The analogue voltage signals were digitalized at 500 Hz and recorded by a Power 1401 digitizer and Spike2 software (CED, Cambridge, UK).

The calcium signals and behavior recordings were both saved for synchronization purposes. Fluorescence signals were acquired and analyzed with MATLAB software. After smoothing the data with a moving average filter (20 ms span), the data was segmented based on behavioral events occurring within individual trials or bouts. The values of fluorescence change (Δ*F*/*F*) were derived by calculating (*F* − *F*_0_)/*F*_0_, where *F*_0_ is the baseline fluorescence signal averaged over a 2‐s‐long control time window. For analyzing the astrocytic calcium responses to open‐arm or closed‐arm entry, the control time window was set 12 s before open‐arm or closed‐arm entry onset to obtain the stable control signals. Δ*F*/*F* values were presented with average plots with a shaded area indicating SEM.

### Two‐Photon Ca^2+^ Imaging

For the two‐photon Ca^2+^ imaging experiments, surgery and recordings were performed as described previously.^[^ ^6c^ ^]^ Briefly, mice were anesthetized with pentobarbital sodium, the brain was removed swiftly and gently, and then acute brain slices containing vHPC region were prepared in ice‐cold oxygenated slicing buffer at the 0.14 mm s^−1^ cutting speed. Slices were incubated at 34 °C in oxygenated ACSF for 30 min and then transferred at room temperature before experiments.

Astrocytes both expressing hM3Dq and GCaMP6m were selected for imaging. Imaging of the slices was performed using an Olympus FV1200MPE two‐photon microscope (Olympus FV1200MPE, Japan) equipped with a 25×, 1.05 NA water‐immersion objective (Olympus, Japan). To locate a target astrocyte, a glass pipette was guided using white light and positioned in proximity to the target. The pipette solution contained either ACSF or ACSF with CNO (5 × 10^−3^ m). Time‐lapse images of the brain slices were analyzed using Fiji software to measure the fluorescence intensity within the region of interest (ROI) in each frame. The change in fluorescence intensity (Δ*F*/*F*) was represented as 100*(*F*_t_ − *F*_0_)/*F*_0_, where *F*_t_ was the fluorescence intensity at time *t* and *F*_0_ was the average fluorescence intensity before the drug application.

### Glutamate, GABA, ATP, and d‐Serine Assay

To measure glutamate, GABA, ATP, and d‐serine levels following SRS, acute hippocampal slices were obtained 24 h after the last restraint stress and incubated in oxygenated ACSF for 10 min, after which the ACSF was collected for analysis. Similarly, to assess glutamate, GABA, ATP, and d‐serine levels after astrocytic activation, acute hippocampal slices from mice expressing hM3Dq were collected 30 min post‐CNO injection and incubated in oxygenated ACSF for 10 min before collection of the ACSF for analysis. For the ATP assay, the ectonucleotidase inhibitor 6‐N, N‐diethyl‐β‐γ‐dibromomethylene‐adenosine‐5‐triphosphate FPL 67156 (ARL 67156 triso‐ dium salt, 100 × 10^−6^ m, Sigma‐Aldrich, #A265) was added to the sample to decrease ATP hydrolysis. All assays were conducted on a 96‐well microplate immediately after sample collection. Glutamate levels were quantified using an assay kit (#MAK004; Sigma‐Aldrich), ATP levels were quantified using an assay kit (#G7571; Promega), GABA levels were quantified using an assay kit (#E‐BC‐K852‐M; Elabscience) and d‐serine levels were measured using an assay kit (#ab241027; abcam), following the manufacturer's instructions.

### Behavioral Tests

All behavioral tests were conducted on male mice between the ages of 8 and 11 weeks unless otherwise specified. Behavioral experiments were conducted during the light cycle, between the hours of 10:00 a.m. and 5:00 p.m.

### Open Field Test

The open‐field chamber utilized in this study measured 50 × 50 cm and was constructed from plastic. It was divided into two areas: a central field (25 × 25 cm) and an outer field (periphery). Individual mice were placed in the center of the field at the beginning of the test and their movements were recorded for a 5‐min period using an automated video tracking system. The path taken by each mouse was digitally captured and analyzed using EthoVision 11.0 software.

### Elevated Plus‐Maze Test (EPM)

The elevated plus‐maze test consisted of four arms measuring 30 × 5 cm, with two open arms lacking walls and two closed arms with 15 × 25 cm high walls. Arms of the same type faced each other. Each mouse was placed in the center of the elevated plus‐maze facing an open arm, and their movements were recorded for a 5‐min period. The amount of time spent in each arm and arm entries were quantified using EthoVision 11.0 software.

### Restraint Stress Mouse Model

For a period of three days of restraint stress, mice were placed in restraint tubes and moved to a separate room from the one used for behavioral experiments. Following a 2‐h (SRS) or 1‐h (ST‐SRS) duration, the mice were taken out of the tubes and carried back to their home cage. The mice in the control group were transferred to a separate room from the one used for anxiety‐like behavioral tests for a duration of 2 or 1 h, without being exposed to restraint stress, and were returned to their home cage subsequently.

### Statistical Analyses

All data are presented as the mean ± SEM. The number of samples examined per each group was specified for each set of data in the corresponding figure caption. Differences between the two groups were determined with Student's *t*‐test or Mann–Whitney *U*‐test depending on its measure and distribution. One‐way ANOVA or two‐way ANOVA followed by the Bonferroni post hoc test was used for multiple group comparisons. Statistical tests were run in GraphPad Prism 8. All statistical data were summarized in Excel as the Supporting Information. The probability value of *p* < 0.05 was considered significant. In the figures, *p*‐values were indicated by asterisk (s): *p < 0.05; **p < 0.01; ***p < 0.001; ****p < 0.0001.

## Conflict of Interest

The authors declare no conflict of interest.

## Author Contributions

J.‐T.L., S.‐Y.J., and J.H. contributed equally to this work. Y.‐H.C., T.‐M.G., and J.‐M.Y. designed the research; J.‐T.L. performed all experiments except for electrophysiological recordings and two‐photon Ca^2+^ imaging with the help of R.‐X.X., J.‐N.X., Z.‐M.L., M.‐L.W., Y.‐W.F., and S.‐H.L.; S.‐Y.J. performed all electrophysiological recordings and analyzed the electrophysiological data; J.H. performed two‐photon Ca^2+^ imaging and analysis; X.‐W.L. provided technical support; J.‐T.L. and J.‐M.Y. interpreted the results with critical input from Y.‐H.C. and T.‐M.G.; J.‐T.L. and J.‐M.Y. assembled the figures and wrote the manuscript.

## Supporting information

Supporting Information

Supplemental Dataset 1

## Data Availability

The data that support the findings of this study are available on request from the corresponding author. The data are not publicly available due to privacy or ethical restrictions.
