# Supplementary material for: Astrocytes in the Ventral Hippocampus Bidirectionally Regulate Innate and Stress‐Induced Anxiety‐Like Behaviors in Male Mice
Source: Adv Sci (Weinh). 2024 Aug 9;11(38):2400354. doi: 10.1002/advs.202400354 (PMC11481230; doi:10.1002/advs.202400354)
Supplement: Supplementary file 1 — Supporting Information [file ADVS-11-2400354-s002.docx]

**Supplemental figures and figure captions**


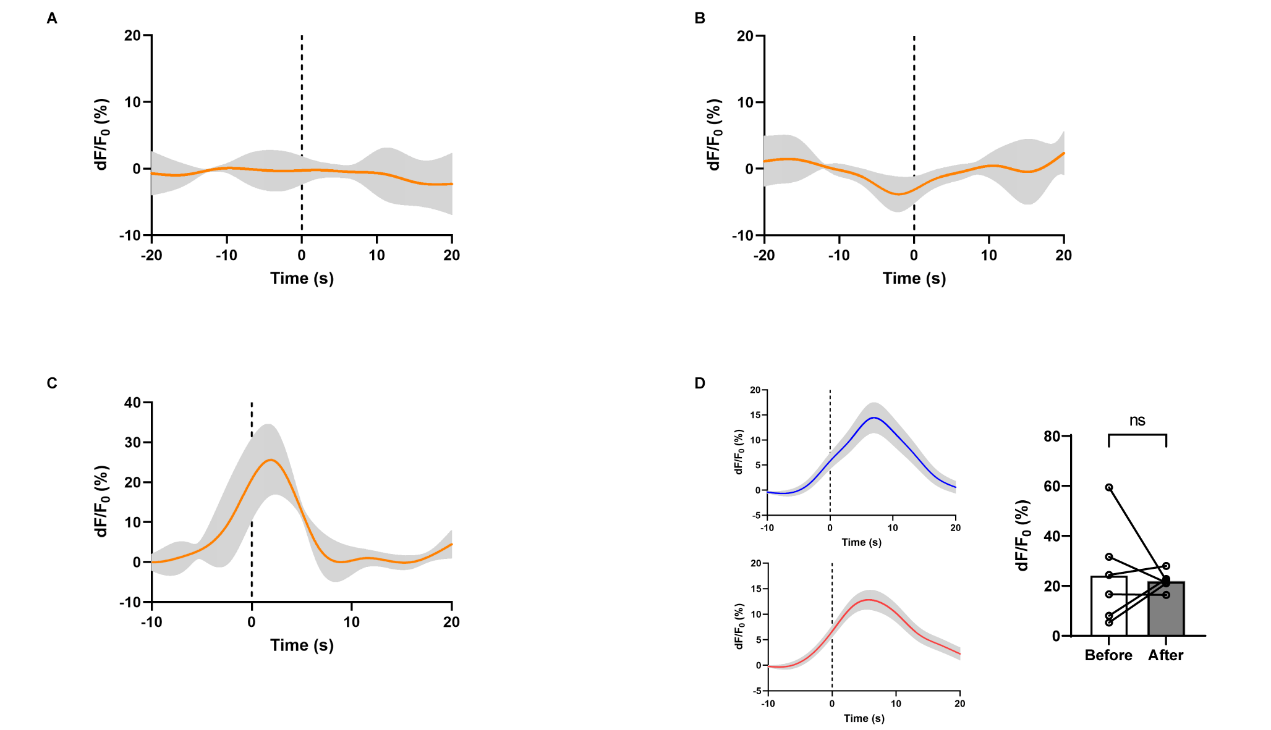


**Figure S1. The activity of ventral hippocampal (vHPC) astrocytes.**

**A** and **B** Representative Ca^2+^ traces during exploration of the open (**A**) or closed (**B**) arms in the elevated plus maze test (EPM) in CKO mice. **C** Same as **A** but for nCKO mice. **D** Representative Ca^2+^ traces (left) and quantification of calcium signals (right) during center exploration in the open field test (OFT) before and after 3-day sub-acute restraint stress (SRS) in control mice. The dotted lines represent the time mice entered the open (**A**, n=6, **C**, n=6) or closed (**B**) arms in the EPM or the center in the OFT (**D**, n=6). Thick blue lines indicate mean and shaded areas indicate SEM. two-tailed unpaired *t* test. ns, no significance. Each data point represents an individual mouse.


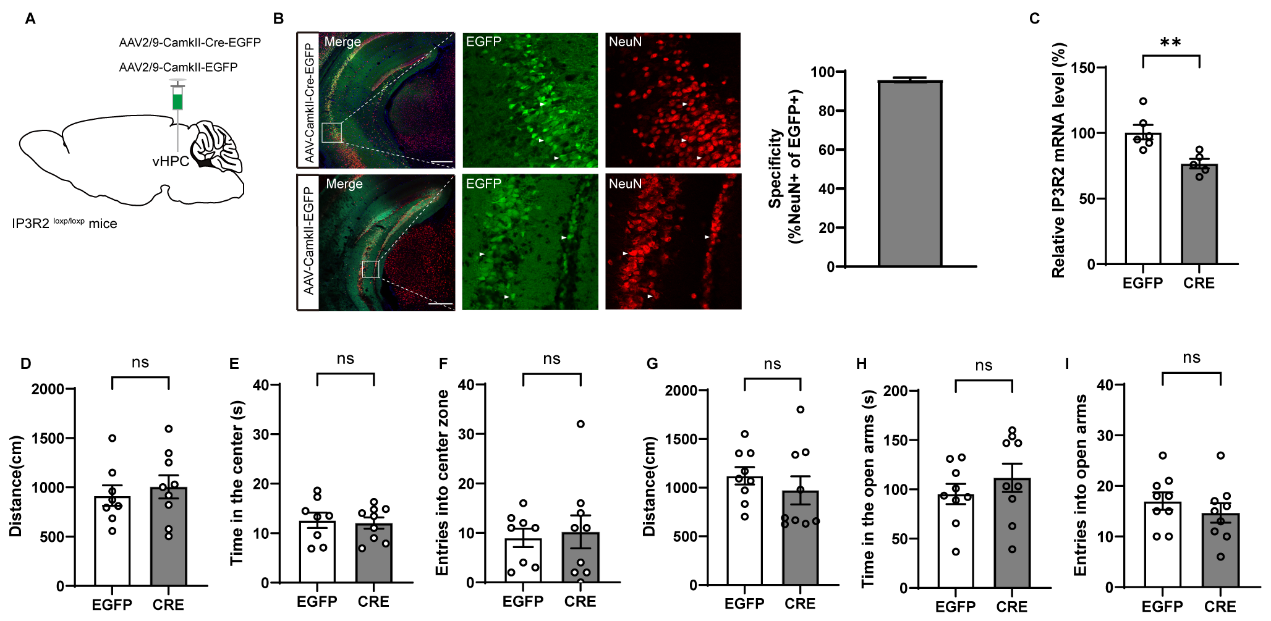


**Figure S2. Conditional neuronal *Itpr2* knockout in the vHPC produced no effects on anxiety-like behaviors.**

**A** and **B** Representative images showing the injection (**A**) and expression (**B**, left) of AAV2/5-CamkII-Cre-EGFP (CRE) and AAV2/5-CamkII-EGFP (EGFP) and quantification of the specificity of Cre recombinase expression (**B**, right) 3 weeks after virus injection in the vHPC of IP3R2^loxp/loxp^ mice. Scale bar, 500 μm. **C** Reduced IP3R2 mRNA level in CRE mice (n=5) compared to EGFP control mice (n=6). **D-F** No significant difference between EGFP group (n=8) and CRE group (n=9) in the OFT. **G-I** No significant difference between EGFP group (n=9) and CRE group (n=9) in the EPM. Data are presented as mean ± SEM; two-tailed unpaired *t* test. ***P* <0.01, ns, no significance. Each data point represents an individual mouse.


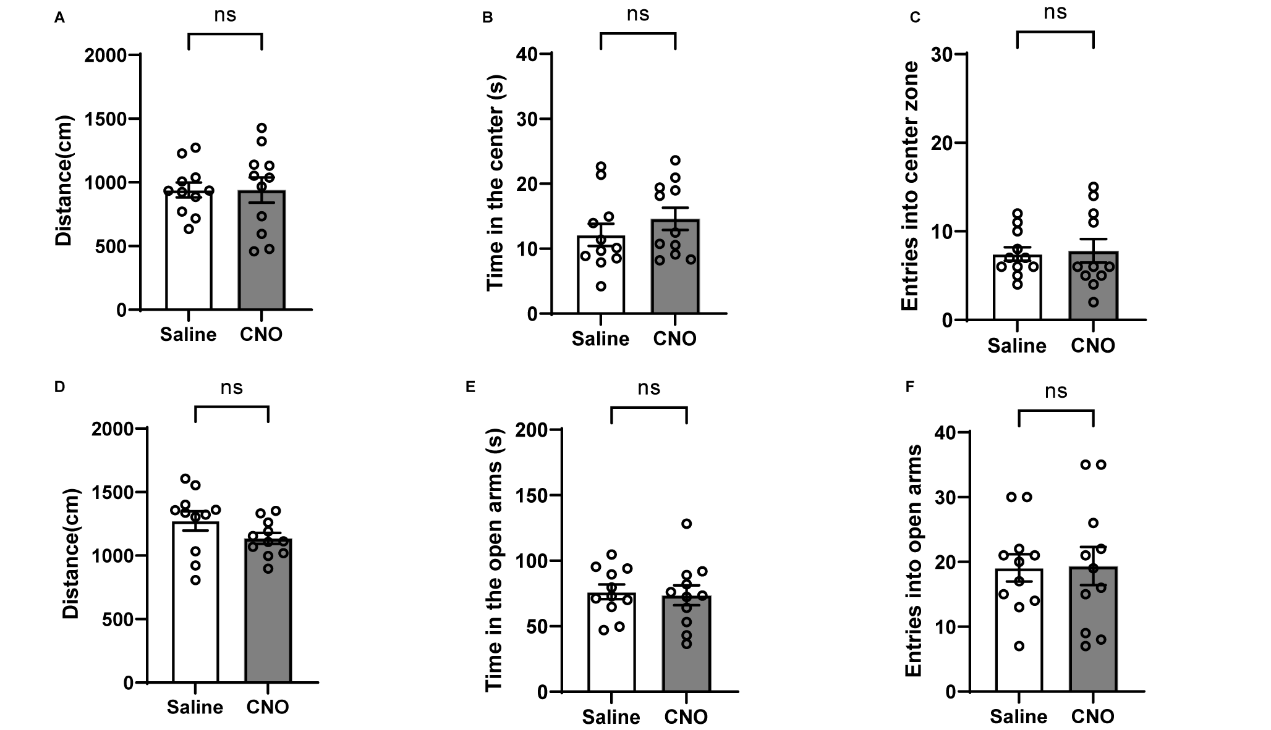


**Figure S3. CNO delivery without virus injection had no impact on anxiety-related behaviors.**

**A-F** No significant difference in the OFT (**A-C**) and EPM (**D-F**) between saline and CNO (3 mg kg^-1^, i.p.) delivery (n=11 mice per group). Data are presented as mean ± SEM; two-tailed unpaired *t* test. ns, no significance. Each data point represents an individual mouse.


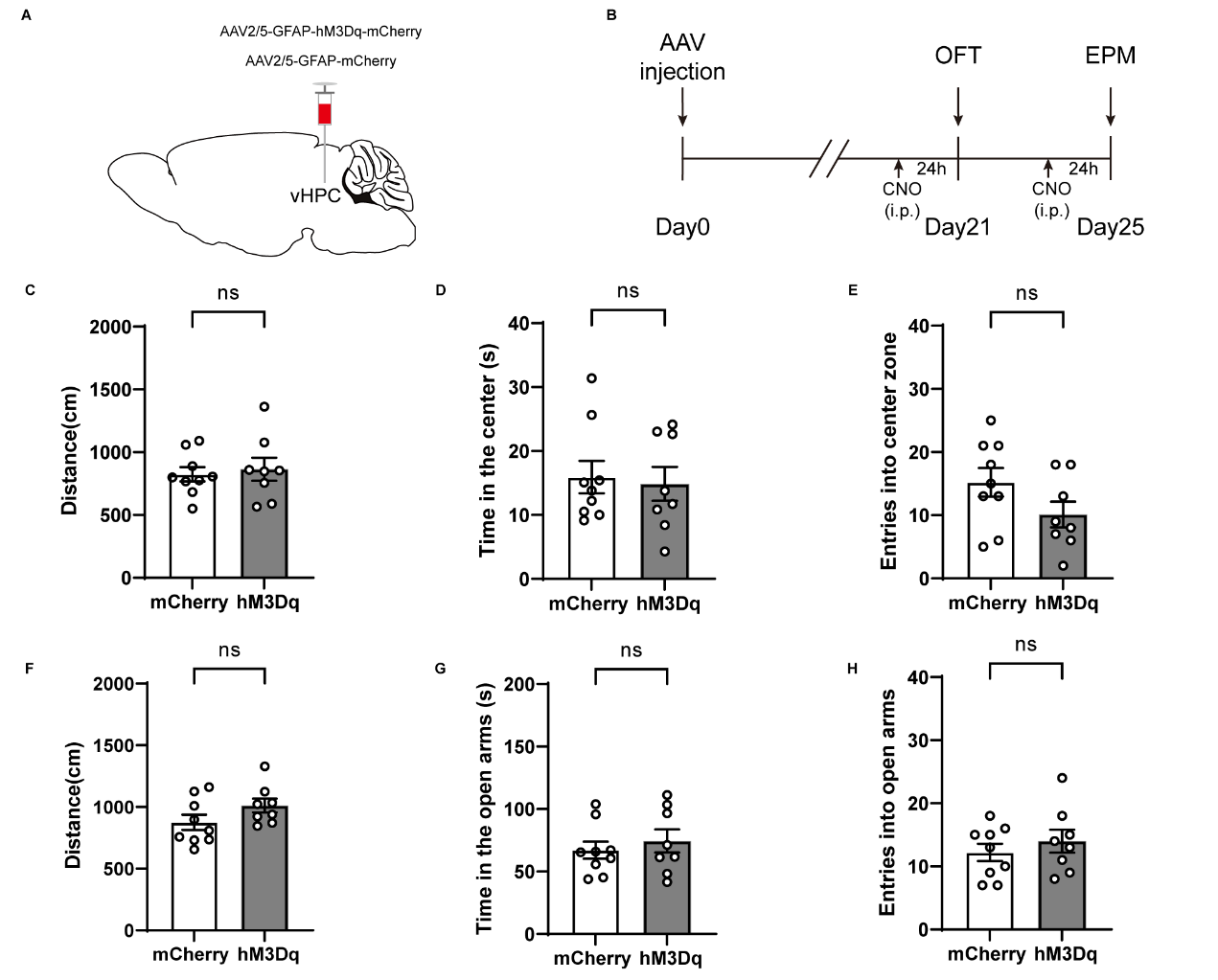


**Figure S4. The anxiogenic effects of chemogenetic activation of vHPC astrocytes were transient.**

**A** and **B** Schematic experimental approach. **C-H** No anxiety-like behavior was observed in the OFT (**C-E**) and EPM (**F-H**) 24 hours after chemogenetic activation of vHPC astrocytes (n=9, 8). Data are presented as mean ± SEM; two-tailed unpaired *t* test. ns, no significance. Each data point represents an individual mouse.


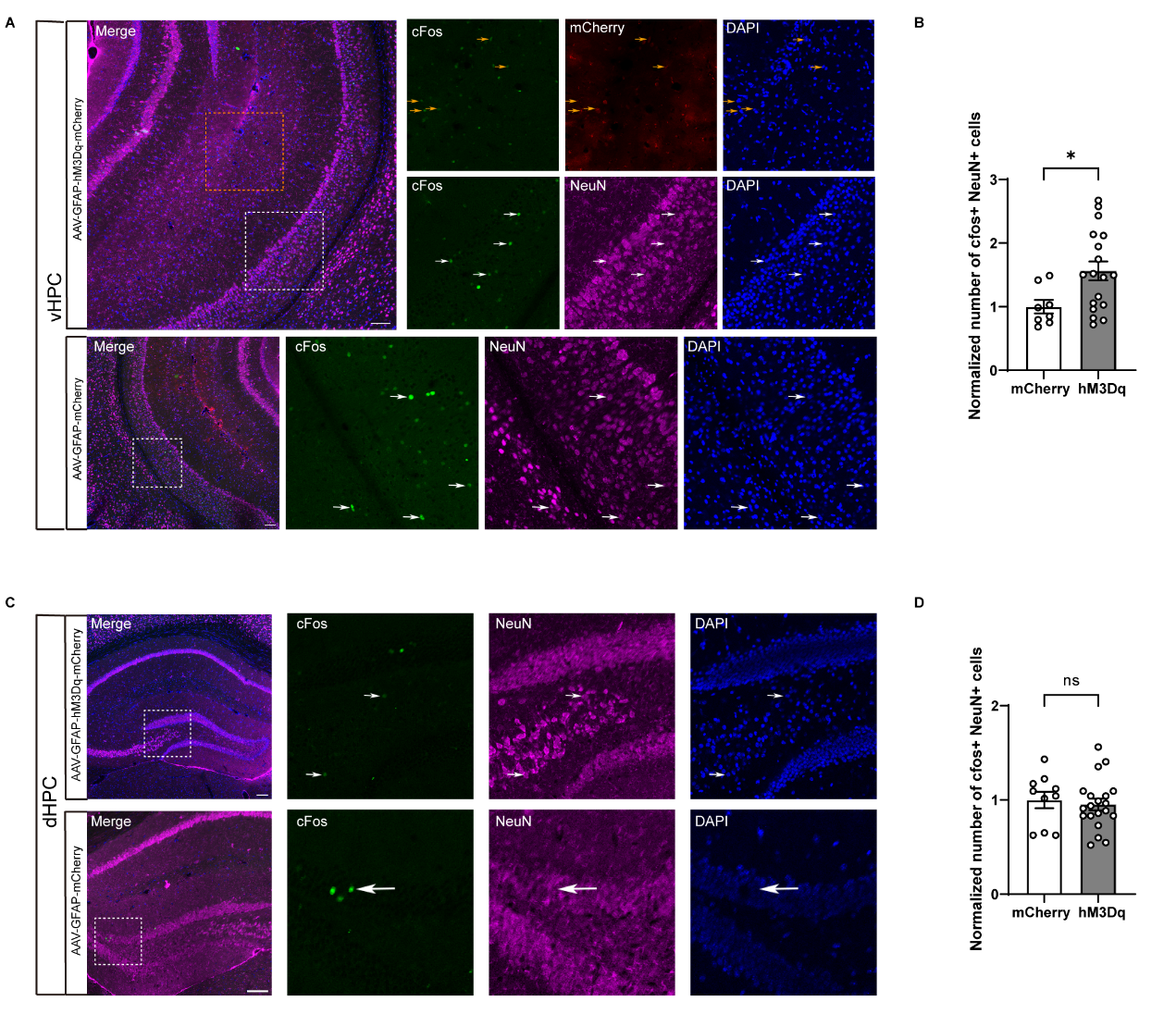


**Figure S5. Expression of c-Fos in the vHPC and dorsal hippocampus (dHPC) after chemogenetic activation of the vHPC astrocytes.**

**A** Representative images showing c-Fos expression in the vHPC after chemogenetic activation of vHPC astrocytes. Yellow arrows indicate astrocytes expressing hM3Dq co-labeled with cFos and GFAP. White arrows indicate neurons expressing NeuN co-labeled with cFos. **B** Number of cFos-expressing neurons (NeuN+) in astrocyte-activated (hM3Dq) group (n=18 from 5 mice) and control (mCherry) group (n=8 from 3 mice). **C** Representative images of c-Fos expression in the dHPC after chemogenetic activation of vHPC astrocytes. White arrows indicate neurons expressing NeuN co-labeled with cFos. **D** Number of cFos-expressing NeuN-labeled neurons in hM3Dq group (n=10 from 3 mice) and mCherry group (n=20 from 5 mice). Scale bar, 100 μm (**A**, **C**). Data are presented as mean ± SEM; two-tailed unpaired *t* test. **P* <0.05, ns, no significance. Each data point represents an individual slice.


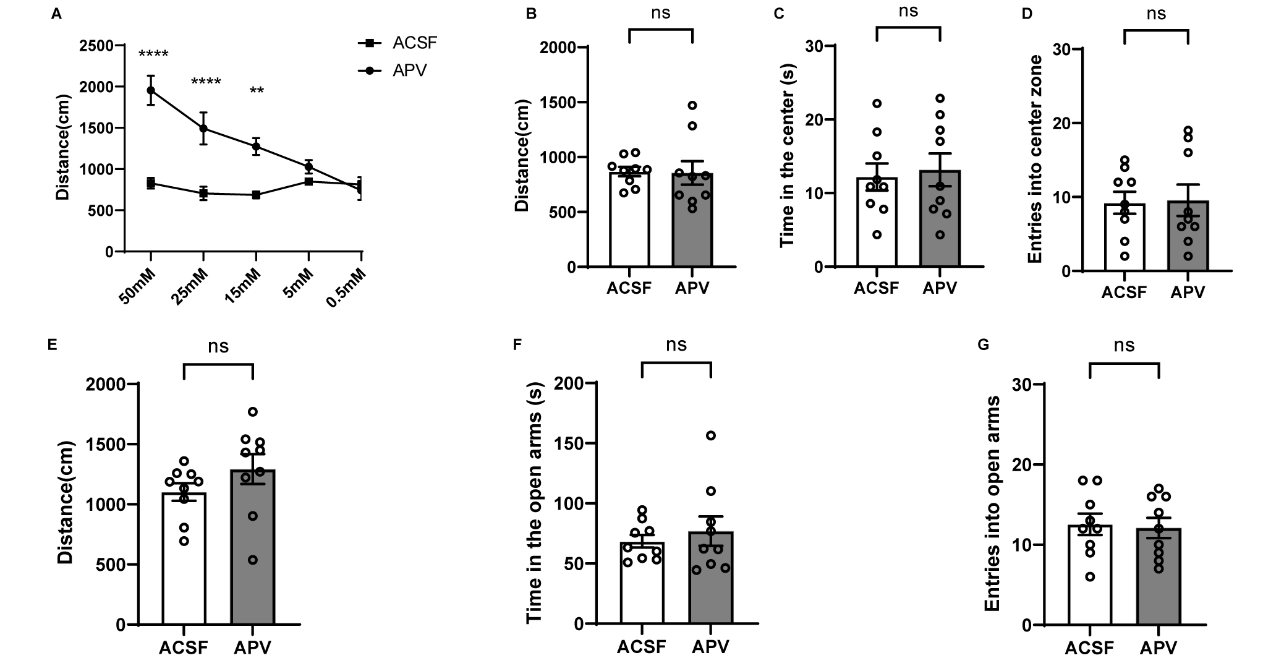


**Figure S6. Local application of APV (5 mM) to the vHPC had no effect on anxiety-like behaviors in mice without hM3Dq expression.**

**A** The effect of different doses of APV on mouse locomotor activity in the OFT. **B-D** OFT. **E-G** EPM. n=9 mice per group. Data are presented as mean ± SEM; two-tailed unpaired *t* test, ns, no significance. Each data point represents an individual mouse.


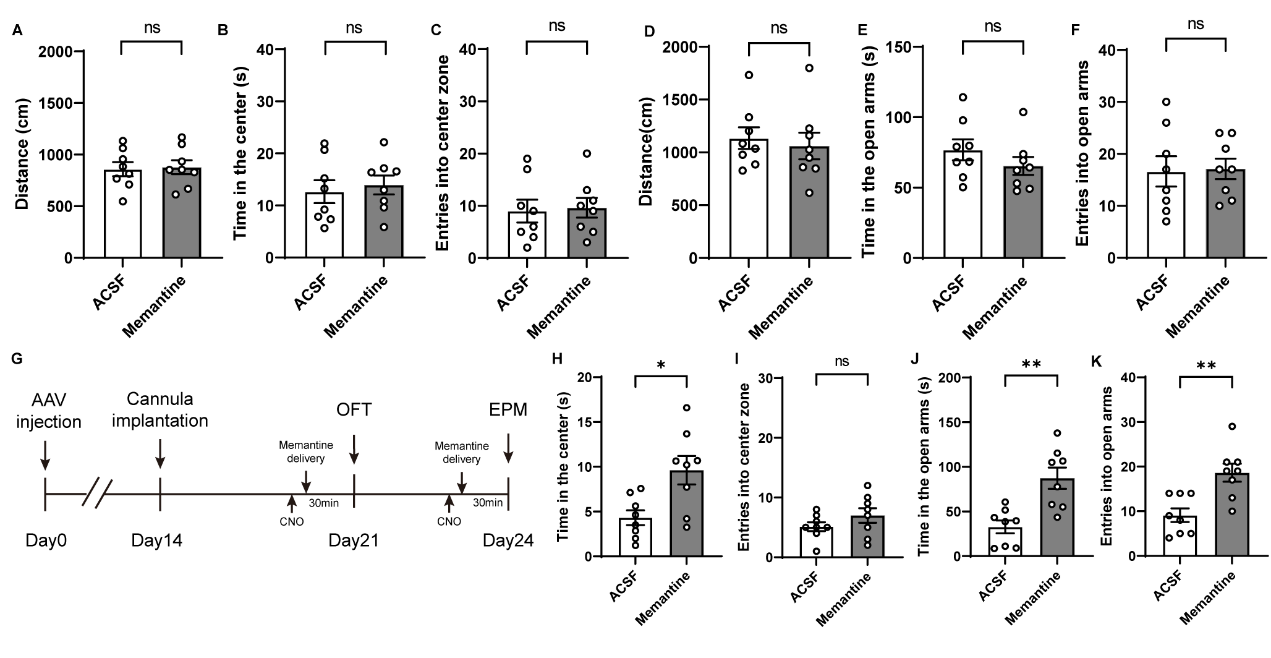


**Figure S7. Local application of memantine (50 mM) to the vHPC had no effect on anxiety-like behaviors** **in stress-naïve mice but prevented anxiety-like behaviors in mice with** **vHPC astrocytes chemogenetical activated.**

**A-F** OFT (**A-C**) and EPM (**D-F**) tests in stress-naïve mice. n=8 for each group. **G** Schematic experimental approach. **H-K** OFT (**H-I**) and EPM (**J-K**) tests in vHPC astrocytes activated mice. n=8 for each group. Data are presented as mean ± SEM; two-tailed unpaired *t* test; **P* <0.05, ***P* <0.01, ns, no significance. Each data point represents an individual mouse.


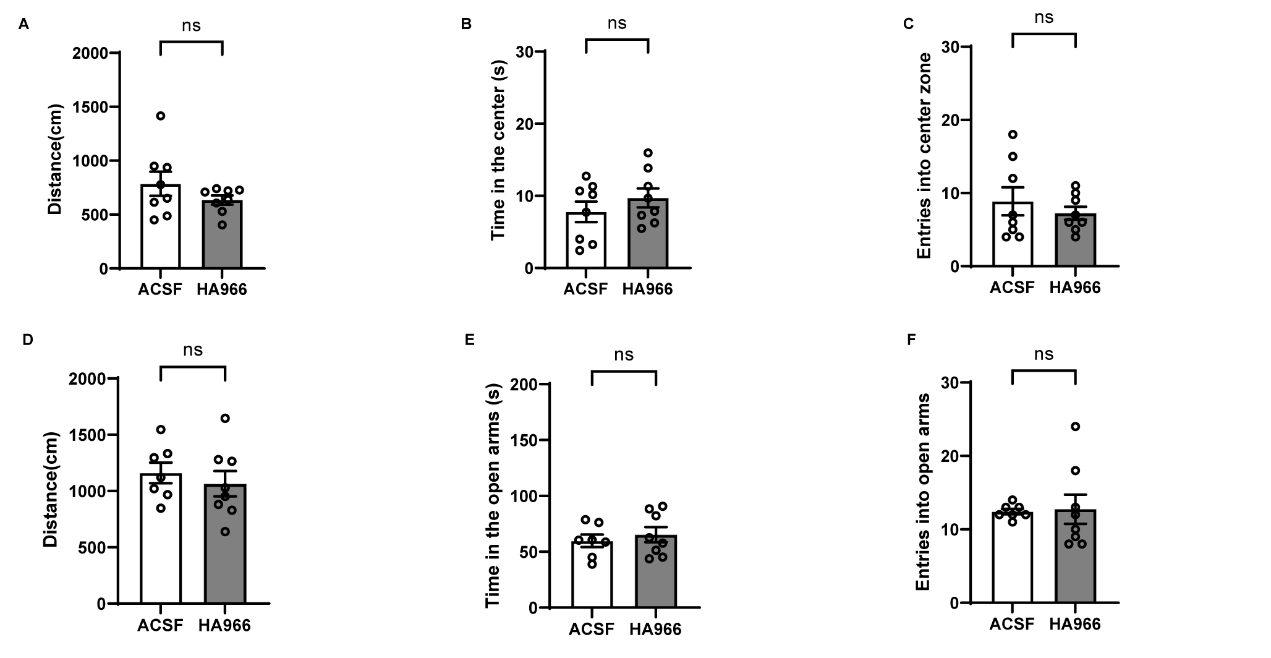


**Figure S8. Local application of (R)-(+)-HA-966 (50 mM) to the vHPC had no effect on anxiety-like behaviors in stress-naïve mice.**

**A-C** OFT (n=8, 8). **D-F** EPM (n=7, 8). Data are presented as mean ± SEM; two-tailed unpaired *t* test, ns, no significance. Each data point represents an individual mouse.


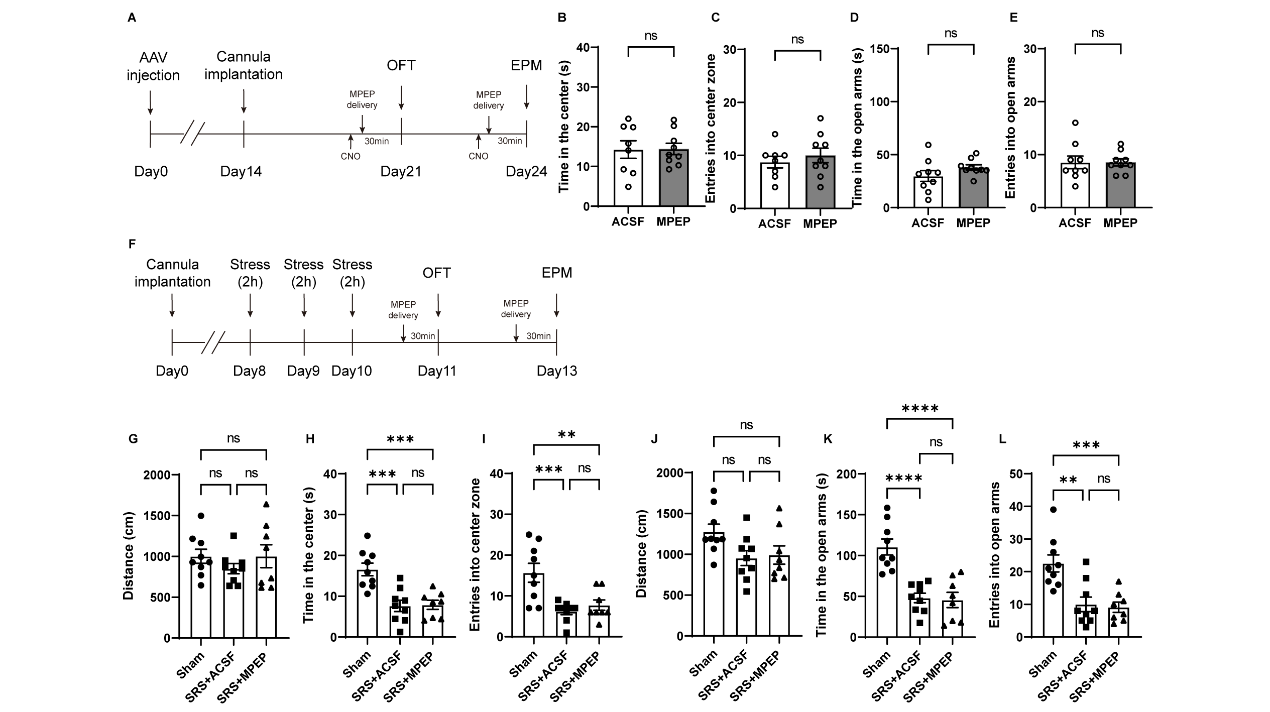


**Figure S9. Local application of the mGluR5 antagonist MPEP (20 μM) to the vHPC failed to prevent the anxiety-like behaviors in vHPC astrocytes activated mice and in mice subjected to SRS.**

**A** Schematic experimental approach. **B-E** OFT (**B, C**, n=8, 9) and EPM (**D, E**, n=9, 9) tests in vHPC astrocytes activated mice. **F** Schematic experimental approach. **G-L** OFT (**G-I**) and EPM (**J-L**) tests in SRS mice. n=9, 9, 8 for each group. Data are presented as mean ± SEM; two-tailed unpaired *t* test (**B-E**), one-way ANOVA followed by the Bonferroni post hoc test (**G-L**). ***P* <0.01, ****P* <0.001, *****P* <0.0001, ns, no significance. Each data point represents an individual mouse.


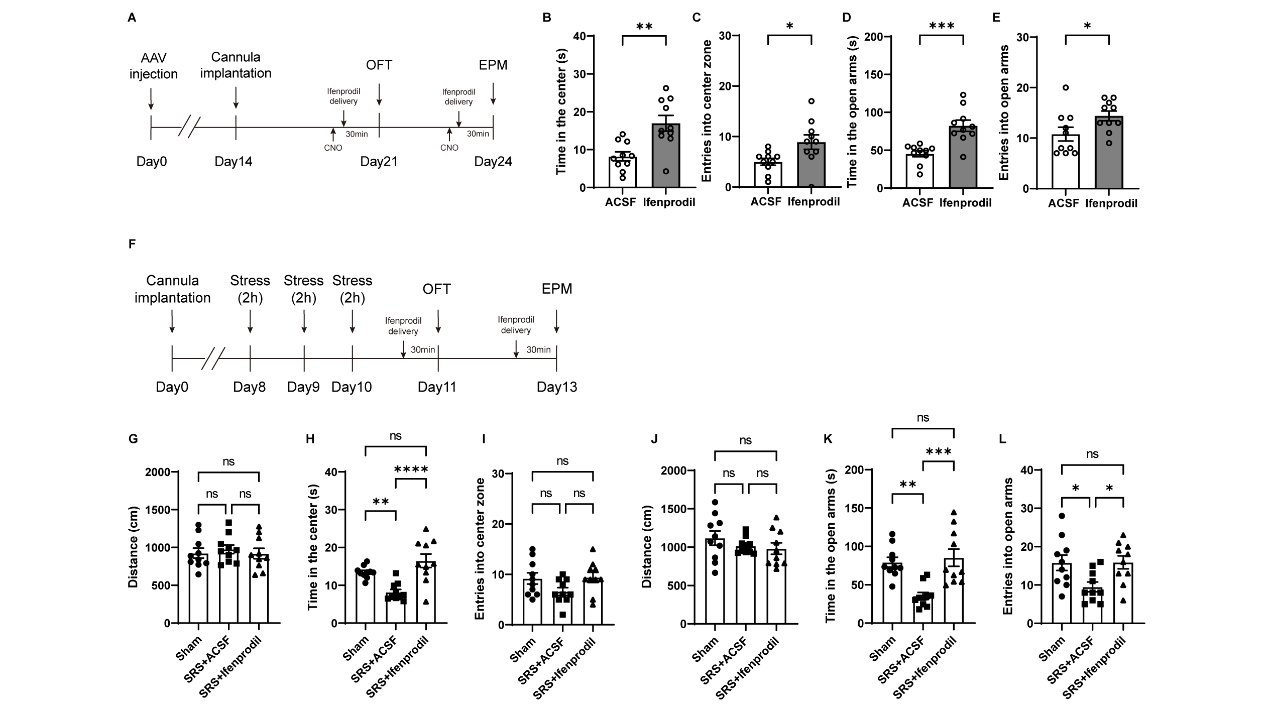


**Figure S10. Local application of the GluN2B-specific antagonist ifenprodil (50 μM) to the vHPC ameliorated the anxiety-like behaviors in vHPC astrocytes activated mice and in SRS mice.**

**A** Schematic experimental approach. **B-E** OFT (**B, C**) and EPM (**D, E**,) tests in vHPC astrocytes activated mice. n=10 for each group. **F** Schematic experimental approach. **G-L** OFT (**G-I**) and EPM (**J-L**) tests in SRS mice. n=10 for each group. Data are presented as mean ± SEM; two-tailed unpaired *t* test (**B-E**), one-way ANOVA followed by the Bonferroni post hoc test (**G-L**). **P* <0.05, ***P* <0.01, ****P* <0.001, *****P* <0.0001, ns, no significance. Each data point represents an individual mouse.


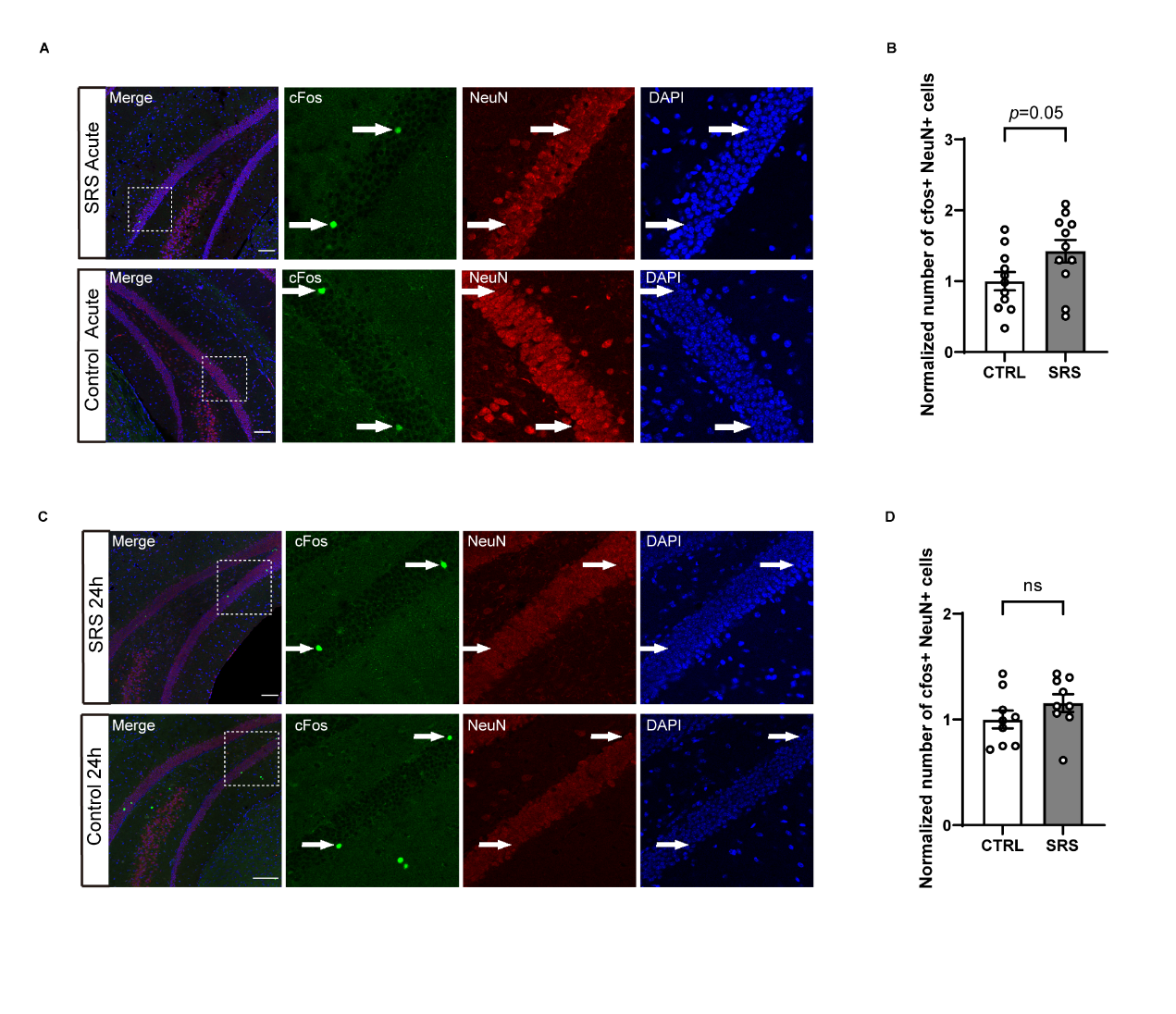


**Figure S11. Expression of c-Fos in vHPC and dHPC neurons after SRS.**

**A** Representative images of c-Fos expression in the vHPC 30 minutes after the last restraint stress of SRS. White arrows indicate neurons expressing NeuN co-labeled with cFos. **B** Number of cFos-expressing NeuN-labeled neurons in control group (n=11 from 4 mice) and SRS group (n=11 from 4 mice). **C** Representative images of c-Fos expression in the vHPC 24 hours after the last restraint stress of SRS. White arrows indicate neurons expressing NeuN co-labeled with cFos. **D** Number of cFos-expressing NeuN-labeled neurons in control group (n=9 from 3 mice) and SRS group (n=9 from 3 mice). Scale bar, 100 μm (**A**, **C**). Data are presented as mean ± SEM; two-tailed unpaired *t* test. **P* <0.05, ns, no significance. Each data point represents an individual slice.


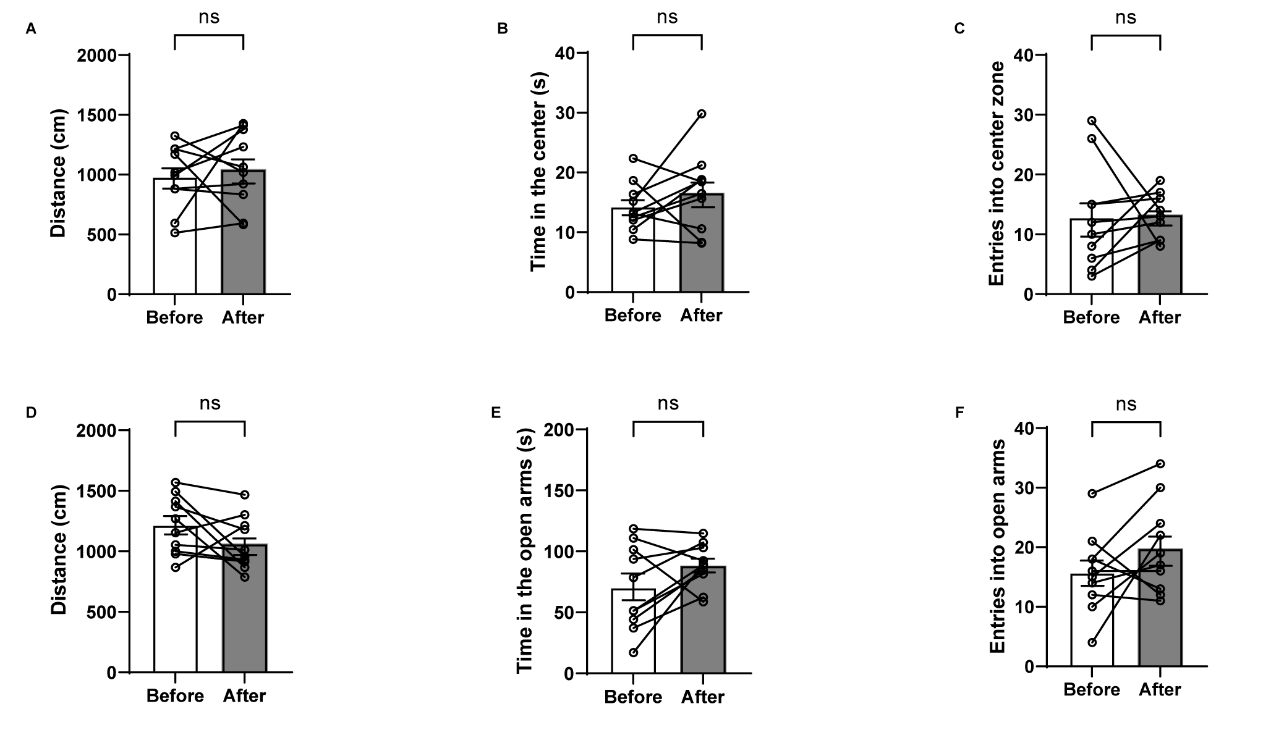


**Figure S12. Anxiety-like behaviors were not altered when mice were retested at a 3-day interval.**

**A**-**C** OFT. n=10 for each group. **D**-**F** EPM. n=10 (**D**), 11(**E**), 12(**F**). Data are presented as mean ± SEM; two-tailed unpaired *t* test. ns, no significance. Each data point represents an individual mouse.


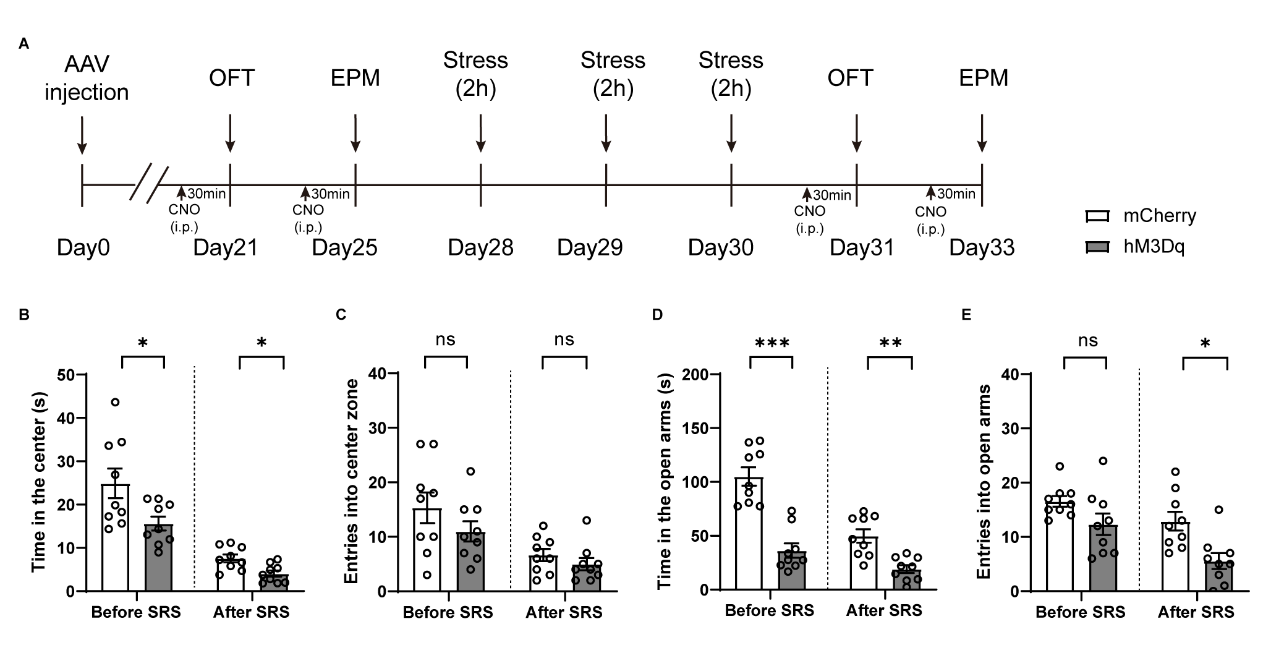


**Figure S13. Chemogenetic activation of vHPC astrocytes deteriorated anxiety-like behaviors in SRS mice.**

**A** Experimental timeline. **B-E** Chemogenetic activation of vHPC astrocytes deteriorated anxiety-like behaviors in the OFT (**G**, **H**, n=9, 9) and EPM (**I**, **J**, n=9, 9) in SRS animals. Data are presented as mean ± SEM. Two-way ANOVA followed by the Bonferroni post hoc test. **P* <0.05, ***P* <0.01, ****P* <0.001, ns, no significance. Each data point represents an individual mouse.


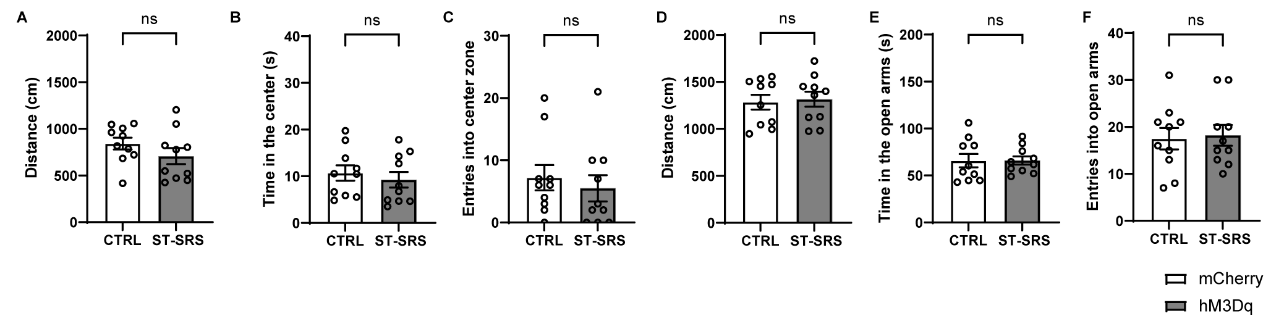


**Figure S14. Mice subjected to subthreshold SRS (ST-SRS,** **1-hour restraint per day for 3 consecutive days) did not display anxiety-like behaviors.**

**A**-**C** OFT. **D**-**F** EPM. n=10 for each group. Data are presented as mean ± SEM; two-tailed unpaired *t* test. ns, no significance. Each data point represents an individual mouse.


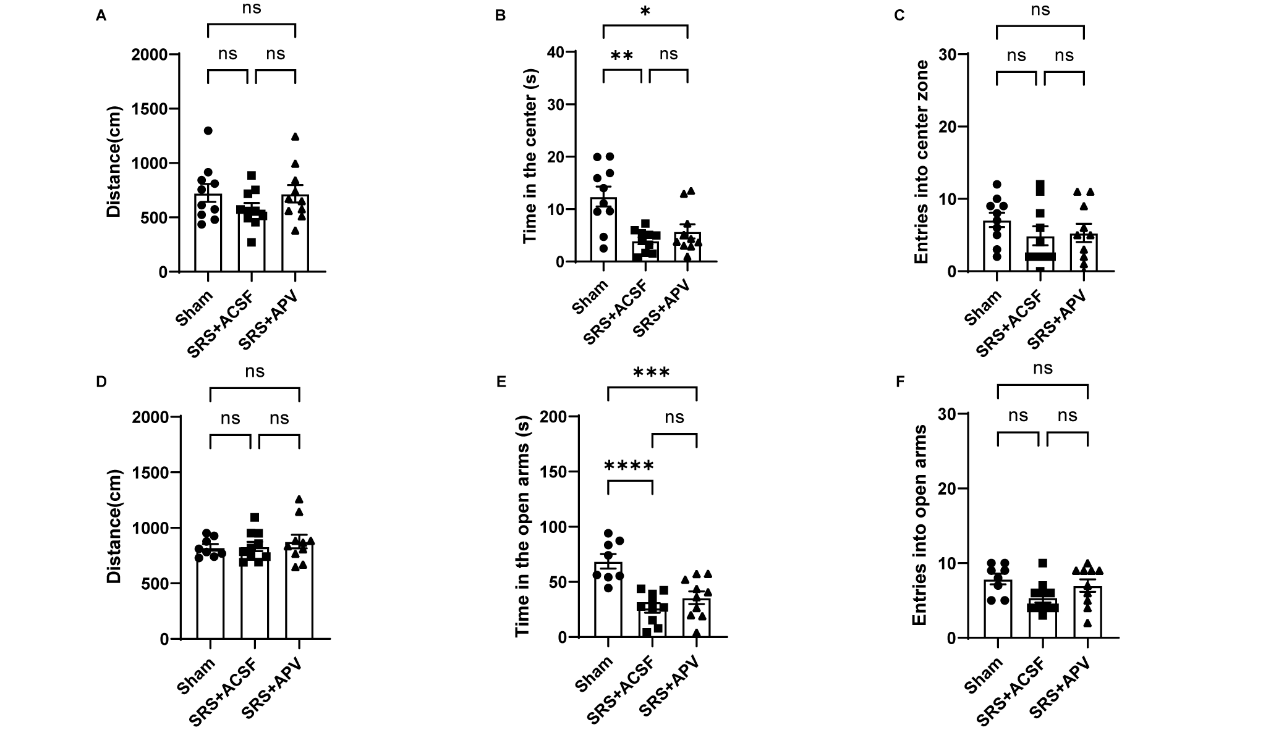


**Figure S15.** **APV-mediated rescue of anxiety-like behaviors caused by SRS disappeared 24 h after delivery.**

**A** No significant difference of distance traveled in the OFT between the three groups (n=10 per group). **B** ACSF- and APV-treated SRS mice spent less time in the center zone than did sham controls. **C** No difference in center zone entries between the three groups. **D** No significant difference of distance traveled in the EPM between the three groups. **E** ACSF- and APV-treated SRS mice (n=10, 10) spent less time in the open arms than did sham controls (n=8). **F** No difference in open arm entries between the three groups. Data are presented as mean ± SEM; one-way ANOVA followed by the Bonferroni post hoc test. **P* <0.05, ***P* <0.01, ****P* <0.001, *****P*<0.0001, ns, no significance. Each data point represents an individual mouse.


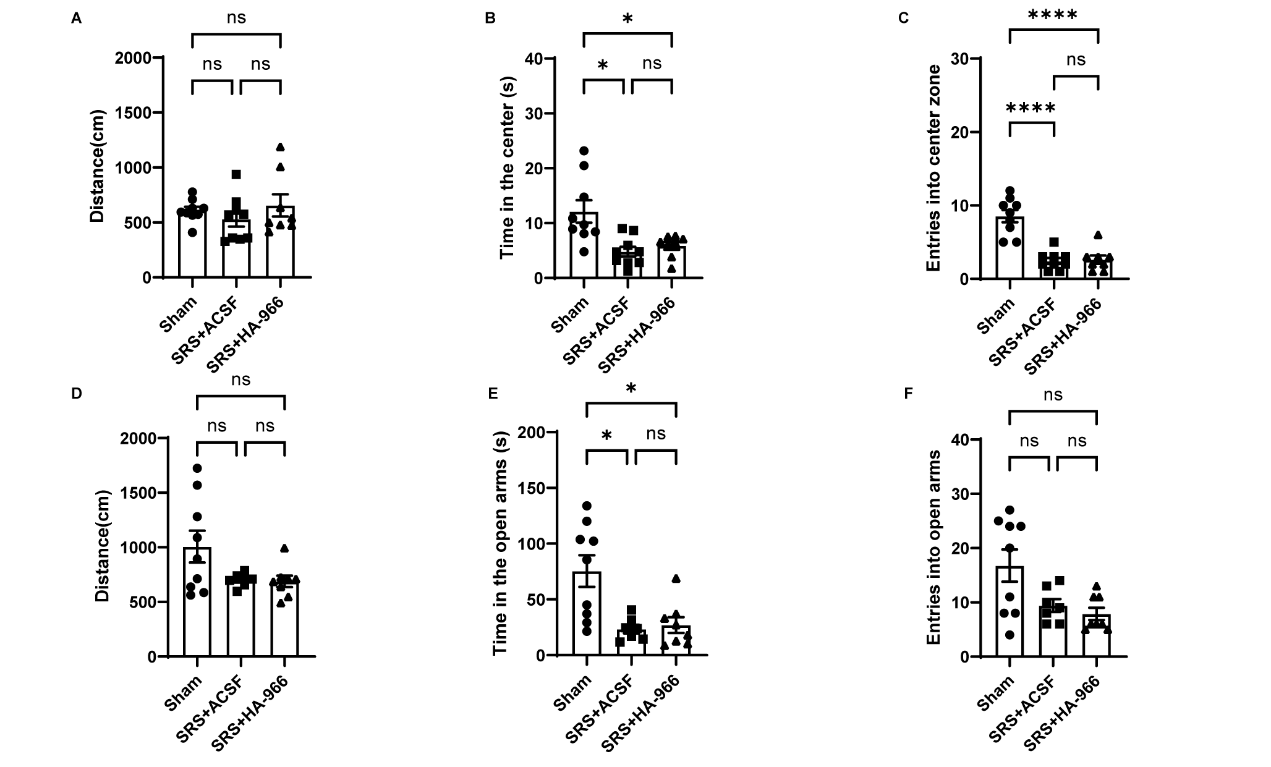


**Figure S16.** **HA966-mediated rescue of anxiety-like behaviors caused by SRS disappeared 24 h after delivery.**

**A-F** ACSF- and HA966-treated SRS mice showed less center zone exploration in the OFT (**A-C**, n=9, 9, 8) and less open arm exploration in the EPM (**D-F**, n=9, 7, 8). Data are presented as mean ± SEM; one-way ANOVA followed by the Bonferroni post hoc test. **P* <0.05, **** *P*<0.0001, ns, no significance. Each data point represents an individual mouse.


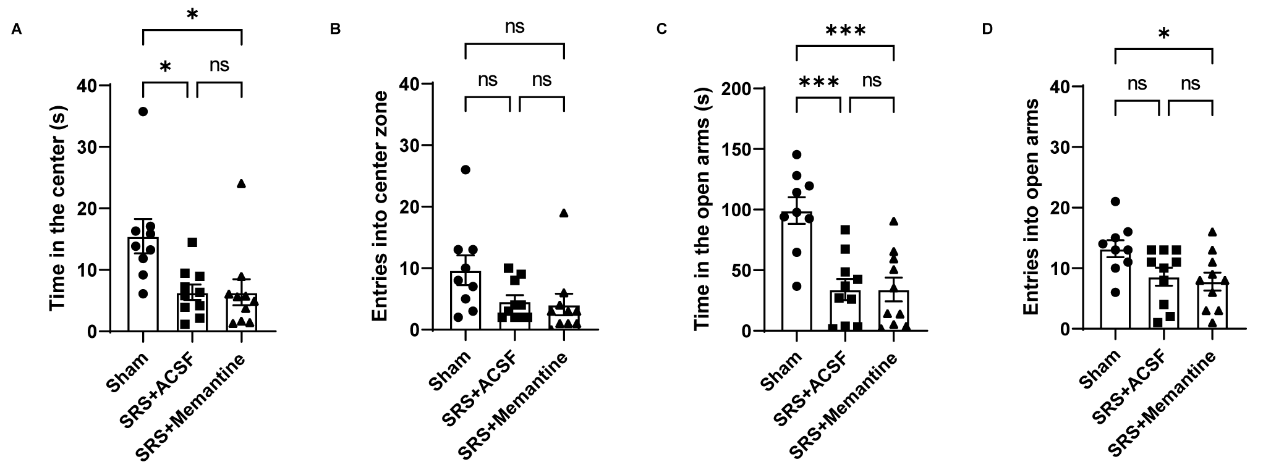


**Figure S17.** **Memantine-mediated rescue of anxiety-like behaviors caused by SRS disappeared 24 h after delivery.**

**A-D** ACSF- and memantine-treated SRS mice showed less center zone exploration in the OFT (**A, B**) and less open arm exploration in the EPM (**C, D**). n=9, 10, 10. Data are presented as mean ± SEM; one-way ANOVA followed by the Bonferroni post hoc test. **P* <0.05, ****P*<0.001, ns, no significance. Each data point represents an individual mouse.


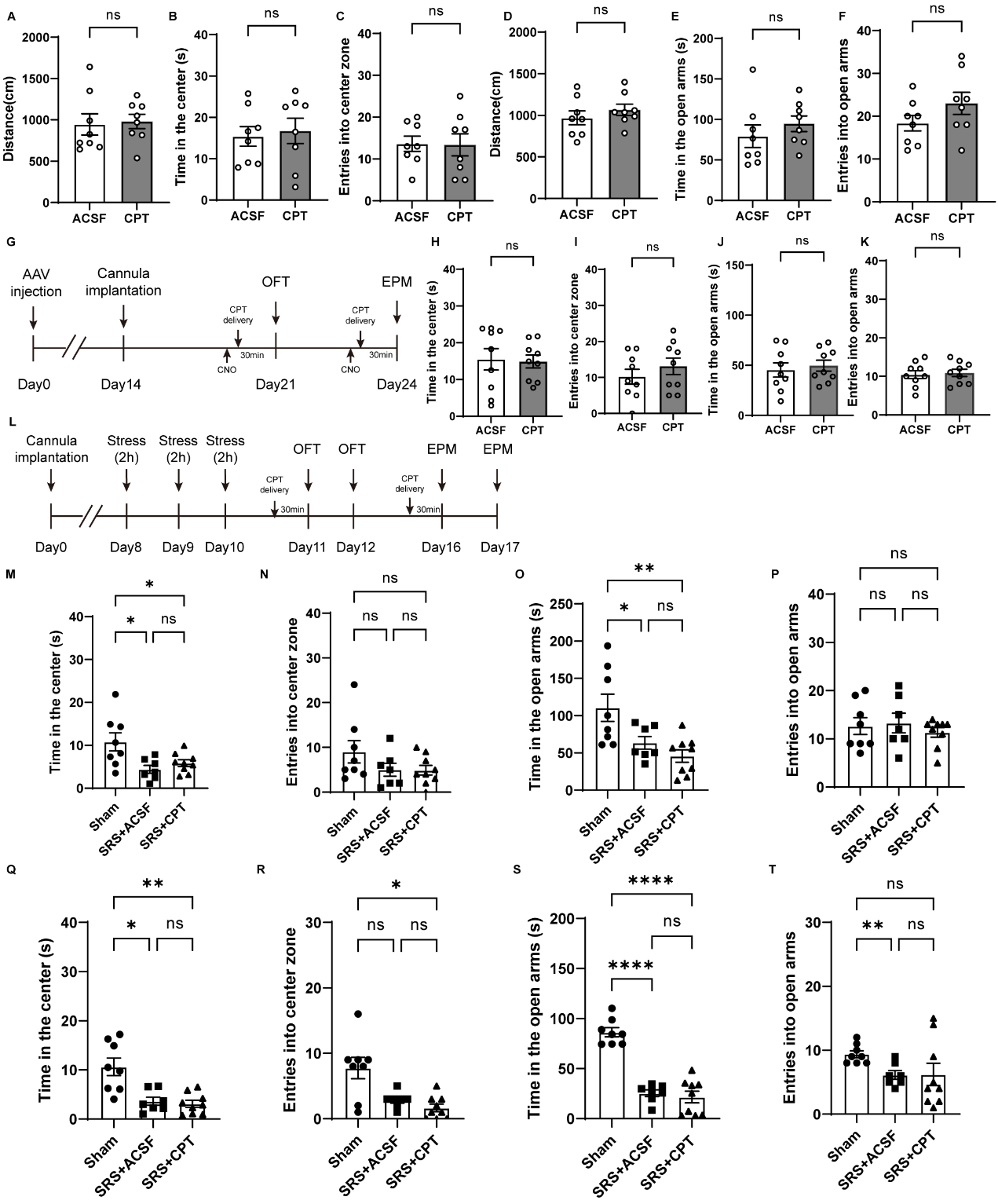


**Figure S18. Local application of adenosine A_1_R antagonist CPT exerted no effect on anxiety-like behaviors in astrocyte-activated mice and in SRS mice.**

**A-F** Local application of CPT (1 mM) to the vHPC had no effect on anxiety-like behaviors in the OFT (**A-C**) and EPM (**D-F**) in stress-naïve mice (n=8 per group). **G** Experimental timeline. **H-K** CPT (1 mM) delivery into the vHPC had no effect on anxiety-like behaviors in the OFT (**H**, **I**) and EPM (**J**, **K**) after vHPC astrocyte activation (n=9 per group). **L** Experimental timeline. **M-P** Local application of CPT (1 mM) to the vHPC had no effect on anxiety-like behaviors in the OFT (**M**, **N**) and EPM (**O**, **P**) in SRS mice (n=8, 7, 9). **Q-T** CPT application had no long-term effect on anxiety-like behaviors in the OFT (**Q**, **R**) and EPM (**S**, **T**) in SRS mice (n=8, 7, 9). Data are presented as mean ± SEM; two-tailed unpaired *t* test (**A-F, H-K**); one-way ANOVA followed by the Bonferroni post hoc test (**M-T**). **P* <0.05, ***P* <0.01, *****P* <0.0001, ns, no significance. Each data point represents an individual mouse.
